# Stepwise assembly of the eukaryotic translation initiation factor 2 complex

**DOI:** 10.1016/j.jbc.2022.101583

**Published:** 2022-01-12

**Authors:** Sven Vanselow, Lea Neumann-Arnold, Franziska Wojciech-Moock, Wolfgang Seufert

**Affiliations:** Department of Genetics, Regensburg Center for Biochemistry, University of Regensburg, Regensburg, Germany

**Keywords:** Cdc123, cell proliferation, eukaryotic initiation factor 2, human eIF2, molecular chaperone, protein assembly, protein complex, protein–protein interaction, translation initiation factor, yeast, co-IP, coimmunoprecipitation, eIF2, eukaryotic translation initiation factor 2, GEF, guanosine exchange factor, GST, glutathione-*S*-transferase, HA, hemagglutinin, Met-tRNA_i_^MET^, methionylated initiator tRNA, TC, ternary complex, WB, Western blot, Y2H, yeast two-hybrid assay

## Abstract

The eukaryotic translation initiation factor 2 (eIF2) has key functions in the initiation step of protein synthesis. eIF2 guides the initiator tRNA to the ribosome, participates in scanning of the mRNA molecule, supports selection of the start codon, and modulates the translation of mRNAs in response to stress. eIF2 comprises a heterotrimeric complex whose assembly depends on the ATP-grasp protein Cdc123. Mutations of the eIF2γ subunit that compromise eIF2 complex formation cause severe neurological disease in humans. To this date, however, details about the assembly mechanism, step order, and the individual functions of eIF2 subunits remain unclear. Here, we quantified assembly intermediates and studied the behavior of various binding site mutants in budding yeast. Based on these data, we present a model in which a Cdc123-mediated conformational change in eIF2γ exposes binding sites for eIF2α and eIF2β subunits. Contrary to an earlier hypothesis, we found that the associations of eIF2α and eIF2β with the γ-subunit are independent of each other, but the resulting heterodimers are nonfunctional and fail to bind the guanosine exchange factor eIF2B. In addition, levels of eIF2α influence the rate of eIF2 assembly. By binding to eIF2γ, eIF2α displaces Cdc123 and thereby completes the assembly process. Experiments in human cell culture indicate that the mechanism of eIF2 assembly is conserved between yeast and humans. This study sheds light on an essential step in eukaryotic translation initiation, the dysfunction of which is linked to human disease.

The translation of mRNAs into proteins marks the final step of gene expression. Because it is a highly energy-intensive and resource-intensive cellular process, it requires a large number of regulating factors to ensure its reliability and accuracy. In eukaryotes, start codons are selected by mRNA scanning. This process is mediated by the 40S ribosomal subunit in collaboration with various protein factors, the eukaryotic translation initiation factors (eIFs) (1, 2). Subsequently, the ribosome subunits join, and the 80S ribosome decodes the ORF of the mRNA. In this stage, termed elongation, a polypeptide chain is synthesized. Translation is completed, and the polypeptide is released after the ribosome recognizes a stop codon.

Eukaryotic translation initiation in particular requires tight control to ensure that the right start codon is recognized with high accuracy. Fundamentally, AUG start codons are recognized by base pairing with the methionylated initiator tRNA (Met-tRNA_i_^MET^). Faithful AUG recognition in addition requires a cascade of interactions between eIFs, the mRNA, and Met-tRNA_i_^MET^ (3, 4). The initiator tRNA is delivered to the ribosomal P site by the trimeric eIF2 complex. During mRNA scanning, Met-tRNA_i_^MET^ is embedded in a ternary complex (TC), which consists of Met-tRNA_i_^MET^, eIF2, and GTP. Interactions between eIF2 subunits with Met-tRNA_i_^MET^, other eIFs, and the mRNAs are crucial for the fidelity of translation initiation. For example, an interaction between the α-subunit of eIF2 and the AUG-3 position enhances translation initiation from start codons in optimal nucleotide context (5, 6). Upon recognition of a start codon, phosphate is released from eIF2 and eIF2-GDP dissociates from the ribosome. Met-tRNA_i_^MET^ remains in the P site, and final steps of translation initiation take place.

In addition to its role as the Met-tRNA_i_^MET^ carrier, eIF2 is a hub for translational regulation and a key target for stress response pathways (7, 8). Cellular stresses, such as amino acid deprivation or viral infection, activate kinases that phosphorylate eIF2α and turn eIF2 into an inhibitor of its own guanosine exchange factor (GEF) eIF2B (9, 10). Consequently, the number of available TCs is decreased, which leads to a depression of global translation. At the same time, translation of specific mRNAs is enhanced. These include *GCN4* in *Saccharomyces cerevisiae* or *ATF4* in mammals. The encoded transcription factors activate the expression of stress adaptation genes.

eIF2 is a heterotrimeric protein complex, which consists of the three subunits α, β, and γ. In yeast, the subunits are named Sui2, Sui3, and Gcd11. eIF2γ is the largest and central protein of the complex. *Via* its domain II, it binds eIF2α domain III. A loop in eIF2γ, comprising amino acids 320 to 335, and the two amino acids, K401 and D403, are of particular importance, according to crystal structures of the archaeal IF2γα dimer (11). Amino acids 248 to 262 and 277 to 301 in domain I of eIF2γ bind to the α1 helix in domain I of eIF2β (11). eIF2γ domain I also contains five conserved GTP-binding motifs and two switch regions, which are important for its function as a small GTPase (12). The N terminus of eIF2γ is variable in length in different species. In *S. cerevisiae*, it seems to fulfill the nonessential function of recruiting PP1 phosphatase to control phosphorylation of eIF2α. In humans, the N terminus is significantly shorter, and PP1 is instead recruited to eIF2α by the PP1 cofactors GADD34 and CReP (13).

Unlike its archaeal counterpart, eukaryotic IF2 requires the dedicated assembly factor Cdc123 to form the heterotrimeric complex (14). The *CDC123* gene was first described in 1984 because of its essentiality for the G1–S cell cycle transition (15). Later, its physical and genetic interactions with eIF2γ were discovered (16, 17), and it was found that the essential function of Cdc123 was in eIF2 assembly (14). Specifically, the association of eIF2γ with eIF2α and eIF2β is dependent on interaction of the eIF2γ C terminus with Cdc123. Indeed, *CDC123* belongs to the group of core essential genes in the human genome, which includes only around 10% of all human genes (18). It is assumed that eIF2 assembly is the only essential function of the *CDC123* gene, as its deletion can be compensated for by combined overexpression of eIF2γ and eIF2α (14). This was also thought to indicate a dependency of eIF2β binding on prior binding of eIF2α. In a crystallographic study, the interaction between eIF2γ and Cdc123 was resolved at the atomic level, which also revealed the membership of Cdc123 in the family of ATP grasp proteins (19). Members of this family of enzymes ligate carboxylic acids to nucleophilic groups; examples are biotin carboxylases, dipeptide synthetases, and tubulin tyrosine ligase (20). However, no enzymatic activity has been identified in Cdc123.

To this date, the exact mode of eIF2 assembly and individual functions of eIF2 subunits have not been investigated thoroughly. In this study, we shed light on individual molecular interactions and propose a detailed model of eIF2 assembly in yeast. It has been shown previously that human Cdc123 can complement Cdc123 in yeast (17, 21). Our study of Cdc123–eIF2 interactions in human cells in addition provides a basis for extrapolating the findings in yeast on eIF2 assembly to higher eukaryotes. In recent years, several related hereditary diseases have been linked to eIF2γ mutations, which affect eIF2 assembly or TC formation (22, 23, 24). Understanding the underlying molecular pathologies could help finding treatments for those medical conditions. Yeast can be a useful model organism for such research, since the mechanism of eIF2 assembly is likely conserved among eukaryotes, as this study indicates.

## Results

### Integrity of the eIF2γ G-domain is vital for eIF2 assembly

**Figure 1:**
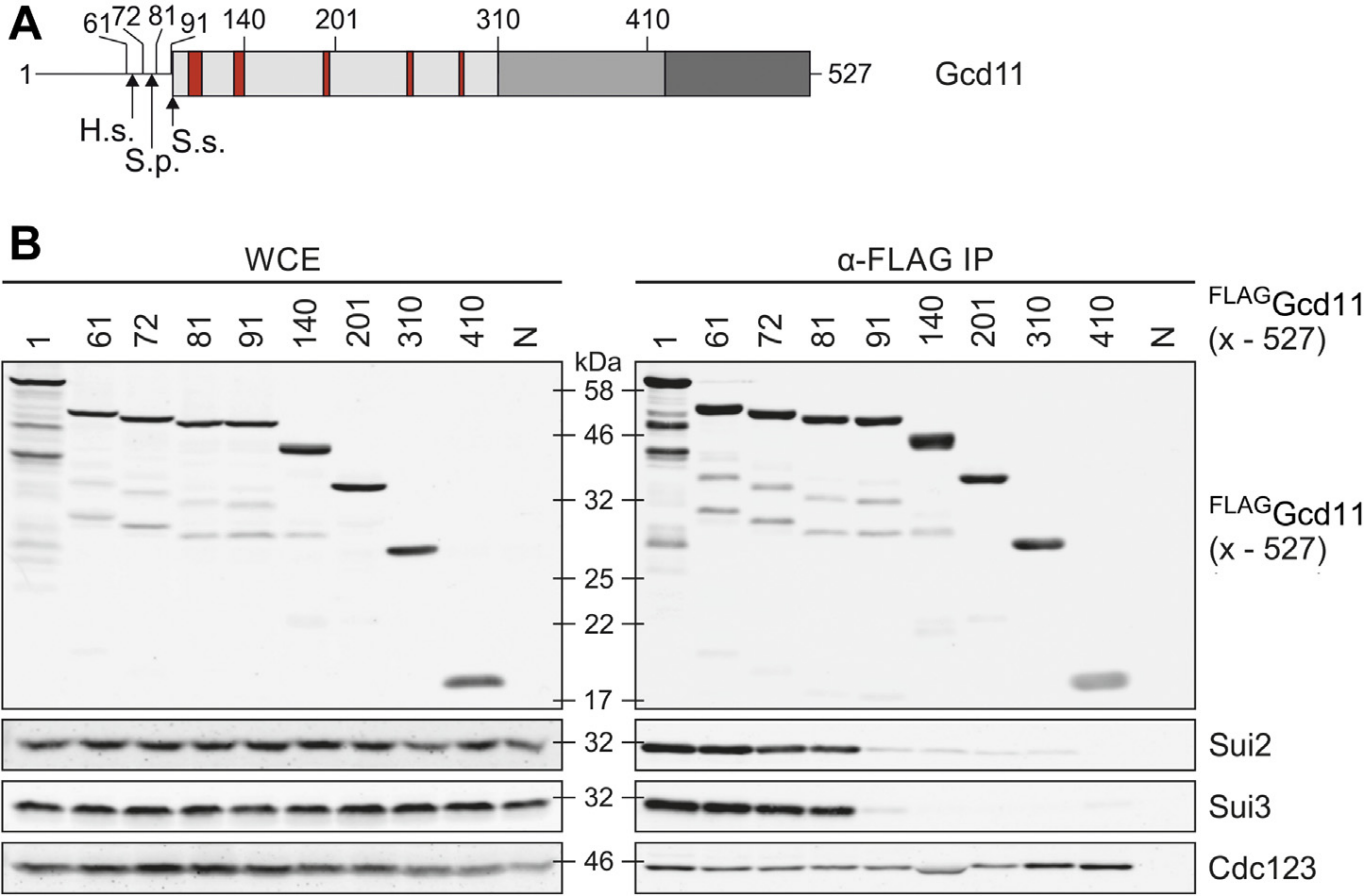
**Role of N-terminal sequences of yeast eIF2γ in eIF2 assembly.***A*, schematic domain structure of yeast eIF2γ (Gcd11). Domain I (amino acids 92–310) is shown in *light gray* including the GTP-binding motifs G–G5 highlighted in *red*, domain II (amino acids 311–421) in *gray*, and domain III (amino acids 422–519) in *dark gray* (11, 12). *Arrows* indicate the eIF2γ N termini in *Homo sapiens* (H.s.), *Schizosaccharomyces pombe* (S.p.), and *Sulfolobus solfataricus* (S.s.). Numbers indicate amino acid positions of N-terminally truncated versions of Gcd11 used for immunoprecipitation shown in (*B*). *B*, WB analysis of interactions between N-terminally truncated and FLAG-tagged variants of Gcd11 and eIF2α (Sui2), eIF2β (Sui3), and Cdc123. Protein levels were analyzed in whole cell extract (WCE, *left panel*) and after anti-FLAG immunoprecipitation (α-FLAG IP, *right panel*). A strain lacking a FLAG epitope (N) was included as specificity control. eIF2, eukaryotic translation initiation factor 2; WB, Western blot.

N termini of IF2γ vary in length between different species (Fig. 1*A*). Compared with orthologs in archaea and other eukaryotes, yeast eIF2γ, encoded by *GCD11*, has an extended N-terminal tail that fulfills a nonessential regulatory function (13). We set out to define the essential parts of eIF2γ needed for eIF2 assembly in yeast. To this end, we created FLAG-tagged versions of Gcd11 with progressively truncated N termini. These variants were expressed in yeast in addition to endogenous Gcd11. We picked strains with similar expression levels for each variant and performed coimmunoprecipitation (co-IP) experiments. The capability of each variant to coprecipitate yeast eIF2α (Sui2), yeast eIF2β (Sui3), and Cdc123 was assessed *via* Western blot (WB) analysis following the IP. Consistent with the nonessential function of the N-terminal extension, removal of the first 60 amino acids did not impair eIF2 assembly. Likewise, truncation up to 81 amino acids resulted in only a mild assembly defect (Fig. 1*B*). However, removal of the first 90 amino acids or more resulted in a drastic reduction of Sui2 and Sui3 binding. Note that the conserved G-domain motifs and known Sui2 and Sui3 binding sequences are contained in variants 91 to 527 (Fig. 1*A*). The concerted loss of interaction with both Sui2 and Sui3 was thus unexpected. These findings suggest that sequences between amino acids 81 and 91 may be critical for G-domain integrity and hint at an interdependency of eIF2α and eIF2β binding to eIF2γ. Domain III of Gcd11 (variants 410–527) was sufficient for Cdc123 binding, consistent with earlier results and the fact that the Cdc123 binding site maps to domain III of eIF2γ (14, 19). Thus, the Cdc123 binding site in domain III is independent of the G-domain, in contrast to the α and β binding sites that require an intact G-domain.

### eIF2α and eIF2β independently bind eIF2γ

**Figure 2:**
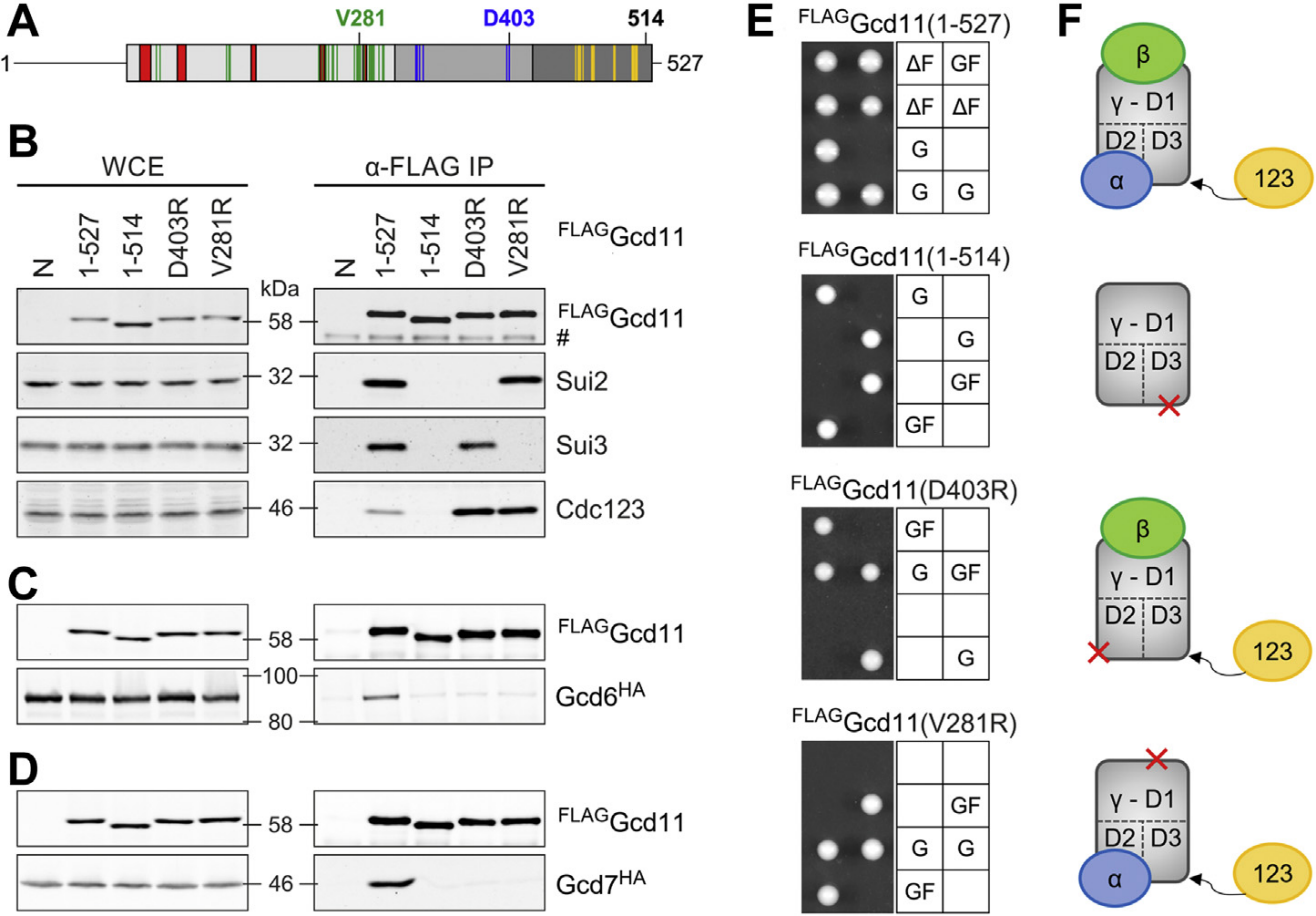
**Independent binding of eIF2α and eIF2β to eIF2γ.***A*, schematic domain structure of yeast eIF2γ (Gcd11) with its binding sites for eIF2α (Sui2), eIF2β (Sui3), and Cdc123. Amino acid positions of Gcd11 involved in binding are highlighted in *blue* for Sui2, *green* for Sui3, and *yellow* for Cdc123 (11, 19, 25). Numbers indicate amino acid positions of mutated and C-terminally truncated versions of Gcd11 used for IP (*B*–*D*) and tetrad dissection (*E*). *B*–*D*, WB analysis of interactions between FLAG-tagged derivatives of Gcd11 with Sui2, Sui3, Cdc123, eIF2Bε (Gcd6), and eIF2Bβ (Gcd7). See Fig. S1 for quantification of coprecipitated Cdc123 by eIF2γ variants. Protein levels were analyzed in WCE (*left panel*) and after α-FLAG IP (*right panel*); the analysis included a no-tag control (N). *E*, tetrad dissection of *gcd11Δ/GCD11* heterozygous diploid strains expressing the indicated ^FLAG^Gcd11 constructs from a gene copy integrated at the *HIS3* locus. For each case, the meiotic progeny of two tetrads is shown. G = endogenous *GCD11*; Δ = *GCD11* deletion allele; F = FLAG-tagged *GCD11* construct. Complementation is indicated by the viability of haploid *gcd11Δ* cells carrying a FLAG-tagged *GCD11* construct, that is, ΔF progeny. No ΔF progeny was observed for the Gcd11 variants 1 to 514, D403R or V281R among more than 20 tetrads analyzed in each case. *F*, schematic model of eIF2 assembly intermediates that can form around Gcd11 variants in *E*. eIF2, eukaryotic translation initiation factor 2; IP, immunoprecipitation; WB, Western blot; WCE, whole cell extract.

The analysis of N-terminally truncated versions of yeast eIF2γ showed combined loss of α-subunit and β-subunit binding (Fig. 1). Since the eIF2α binding sites are found in domain II of eIF2γ, this may indicate a dependency of eIF2α binding on eIF2β or possibly an interdependency. This idea is consistent with the previously observed rescue of a Cdc123-deficient strain by the combined overexpression of eIF2α and eIF2γ (14). To address this possible interdependency, we aimed to create variants of yeast eIF2γ (Gcd11) with disruptive amino acid exchanges in the eIF2α or eIF2β binding platforms (Fig. 2*A*). The residues V281 and D403 were chosen based on crystal structures of archaeal IF2 (25, 26). D403 interacts with an amide group in domain III of eIF2α, and its acidic character is conserved in all known IF2γ sequences. V281 is found in a β-sheet at the end of domain I, close to the G5 motif, and interacts with eIF2β. The nonpolar nature of the residue is conserved in archaea and eukaryotes. Both amino acids were mutated to arginine, a large and basic amino acid, to disrupt subunit binding. The Gcd11 variants 1 to 514 was included as a control. This truncation derivative of yeast eIF2γ lacks sequences close to the binding site for Cdc123, fails to bind Cdc123, and consequently fails to associate with the α-subunits and β-subunits (14, 19). As before, we coexpressed the variants with endogenous Gcd11, selected strains with similar expression levels, and tested for interaction with Sui2, Sui3, and Cdc123 in co-IP experiments. As observed previously, Gcd11 (1–514) failed to coprecipitate detectable levels of Sui2, Sui3, and Cdc123 (Fig. 2, *B* and *F*). However, the variants D403R and V281R, while unable to interact with either Sui2 or Sui3, showed largely intact binding of the respective other subunit (Fig. 2, *B* and *F*). This indicates that binding of eIF2α and eIF2β to eIF2γ is largely independent of each other so that either subunit can form a dimeric complex with eIF2γ.

Interestingly, the V281R and D403R mutants seemed to associate with Cdc123 more stably than WT Gcd11, similar to some Gcd11 versions lacking the G-domain (Fig. 1*B*). Cdc123 binding was quantified for both mutants and variants 1 to 514. The binding mutants indeed coprecipitated higher amounts of Cdc123 than WT Gcd11, in particular variant D403R (D403R: 537 ± 112%; V281R: 317 ± 2%; n = 3; Fig. S1*A*). We concluded that each of the mutants can form a specific eIF2 assembly intermediate in which Cdc123 is still attached and unable to dissociate. This hypothesis was supported by cell growth experiments, where the genes of Gcd11 variants were put under control of the inducible *GALL* promoter (27). Diploid and haploid strains were used to compare differential expression levels. Cell viability under noninducing and inducing conditions was tested in a spot dilution assay (Fig. S1*B*). The test was also performed in cells simultaneously overexpressing Cdc123. We observed a dominant negative effect on cell viability for variant D403R, which does not bind eIF2α. Cell viability was restored by overexpression of Cdc123. We then repeated the test in haploid cells, where the relative gene dose is presumably twice as high as in the diploid cells, to assess the dose-dependency of the effect. Here, variant D403 showed an even stronger effect on cell viability, and a minor effect was also observed for variant V281R, which does not bind eIF2β. Similar expression of all Gcd11 constructs was verified by WB analysis (Fig. S1*C*). The effect thus correlates directly with the extent of Cdc123 binding and is dose dependent. It seems that Gcd11 variants that are unable to complete assembly permanently bind Cdc123, thus limiting the pool of available Cdc123 and inhibiting translation.

Next, we analyzed the capacity of each Gcd11 variant to interact with eIF2B. To this end, yeast strains with endogenously hemagglutinin (HA)-tagged eIF2B subunits β (Gcd7) or ε (Gcd6) were crossed with Gcd11 variant expressing strains and subjected to co-IP. The interaction between partially assembled eIF2 and eIF2B would likely be significantly weakened because all eIF2 subunits contact eIF2B *in vivo* (28). As expected, a strong interaction with Gcd6 and Gcd7 was observed for WT Gcd11. For the mutants, we did not detect any coprecipitated Gcd6 or Gcd7 (Fig. 2, *C* and *D*), which indicates that dimeric eIF2γα and eIF2γβ complexes fail to associate with eIF2B.

Finally, we tested the ability of all variants to complement deletion of the endogenous *GCD11* gene in yeast. The FLAG-tagged variants were expressed in *gcd11Δ/GCD11* heterozygous diploid strains. Strains were sporulated, and meiotic progeny was investigated by tetrad dissection. Unsurprisingly, ^FLAG^Gcd11 WT restored cell viability of haploid cells lacking endogenous *GCD11*. On the other hand, none of the variants complemented the *GCD11* gene deletion (Fig. 2*E*). Thus, dimeric eIF2 complexes lacking α or β do not provide eIF2 function *in vivo*.

### eIF2α and eIF2β have distinct roles in eIF2 assembly

**Figure 3:**
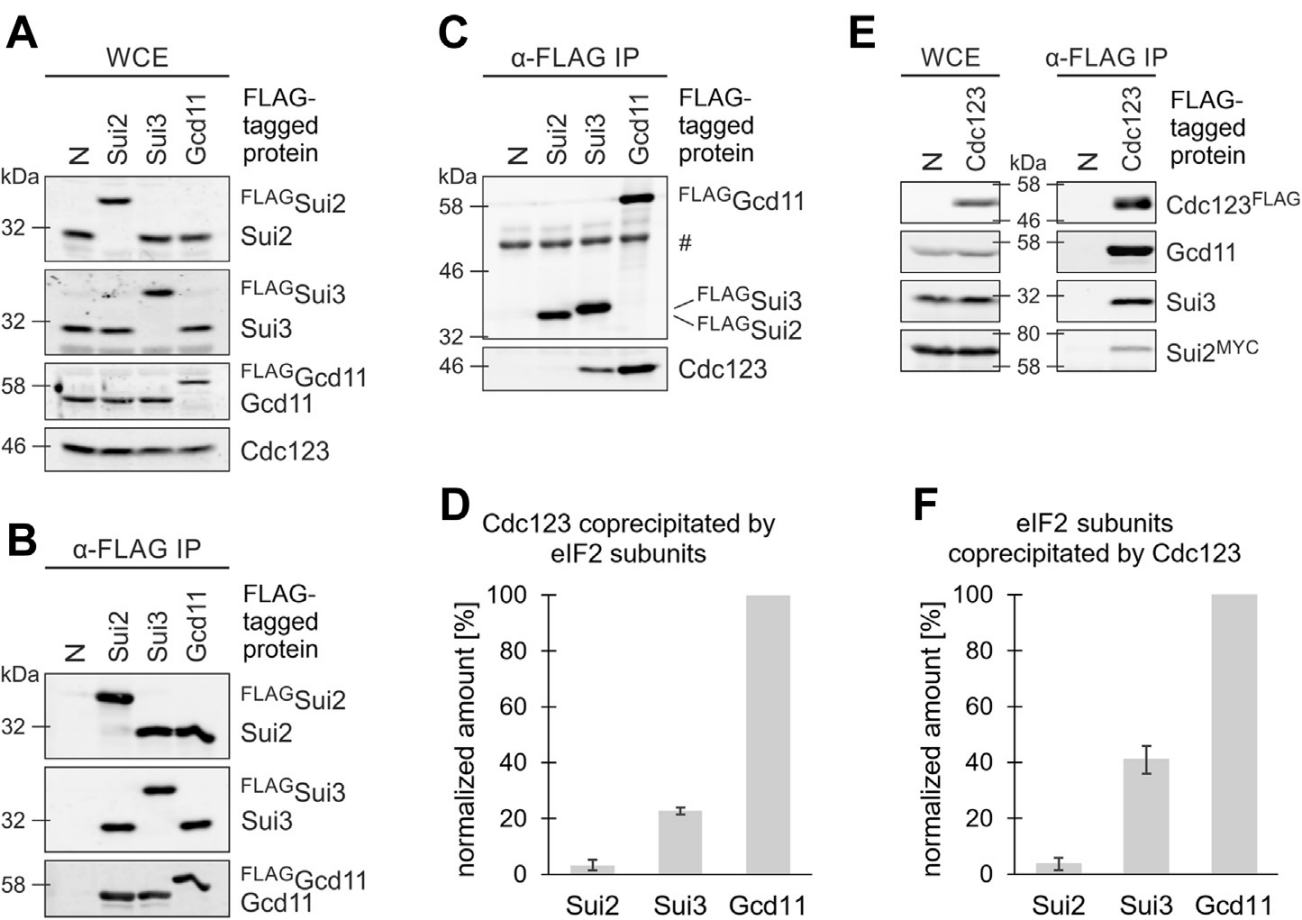
**Quantitative analysis of eIF2 assembly intermediates.***A* and *B*, Sui2, Sui3, or Gcd11 was endogenously FLAG tagged and analyzed for expression levels (*A*) and integration into eIF2 complexes (*B*). *C* and *D*, WB quantification of ^FLAG^eIF2 subunits and coprecipitated Cdc123. # indicates immunoglobulin G (IgG) heavy chain. Signals of immunoprecipitated eIF2 subunits and coprecipitated Cdc123 were quantified by use of an infrared imaging system. Cdc123 signals were normalized to the amount of the corresponding ^FLAG^eIF2 subunit in IP samples. No-tag control (N) served as blank value. The coprecipitation of Cdc123 is shown as mean and SD, and coprecipitation of Cdc123 by Gcd11 was set to 100% (n = 3). *E* and *F*, WB analysis of interaction between Cdc123^FLAG^ and eIF2γ (Gcd11), eIF2β (Sui3), and eIF2α (Sui2^MYC^). Protein levels were analyzed in WCE (*left panel*) and after α-FLAG IP (*right panel*); the analysis included a no-tag control (N). Signals of immunoprecipitated Cdc123 and coprecipitated eIF2 subunits were quantified by use of an infrared imaging system. eIF2 subunit signals in the IP sample were normalized to the signal in WCE samples. The coprecipitation of eIF2 subunits is shown as mean and SD. Coprecipitation of Gcd11 was set to 100% (n = 3). eIF2, eukaryotic translation initiation factor 2; IP, immunoprecipitation; WB, Western blot; WCE, whole cell extract.

To further analyze the eIF2 assembly pathway, we aimed to quantify naturally occurring eIF2 assembly intermediates. To this end, we created yeast strains in which a FLAG epitope sequence was fused to the endogenous *SUI2*, *SUI3*, or *GCD11* genes. The resulting strains grew at the rate of the untagged WT strain (Fig. S2). WB analysis with subunit-specific antisera indicated that the FLAG-tagged versions were expressed at physiological levels (Fig. 3*A*). Moreover, co-IP analysis confirmed regular incorporation of the FLAG-tagged subunits into eIF2 complexes (Fig. 3*B*). FLAG immunoprecipitates were also analyzed for the presence of the assembly factor Cdc123. To quantify the associations, Cdc123 signals were normalized to the signal corresponding to each ^FLAG^eIF2 subunit, and the experiment was performed in triplicates. Gcd11 coprecipitated the highest amount of Cdc123, consistent with previous data (14). In addition, we detected moderate amounts of a Sui3–Cdc123 complex (23 ± 1% compared with Gcd11–Cdc123) and very low amounts of Sui2–Cdc123 (3 ± 2%; Fig. 3, *C* and *D*). To substantiate these findings, we reversed the experimental setup and used C-terminally FLAG-tagged Cdc123 to quantify coprecipitation of eIF2 subunits. Sui2 was tagged with a C-terminal 13xMYC epitope in anticipation of a weak signal. We quantified the enrichment of eIF2 subunit signals from whole cell extract to IP in triplicates. Cdc123^FLAG^ precipitates contained high amounts of Gcd11, lower amounts of Sui3, and little Sui2^MYC^ (Gcd11: 100%, Sui3: 41 ± 5%, and Sui2: 4 ± 2%; Fig. 3, *E* and *F*). The relative proportions of Cdc123–eIF2 subunit complexes conformed to the results from the previous experiment.

Together, these data provide evidence for an abundant dimeric Cdc123–Gcd11 intermediate. Assembly intermediates containing Sui3 are less abundant, whereas Sui2 containing intermediates are hardly detectable. The significant quantitative difference between Cdc123–Sui2 and Cdc123–Sui3 complexes hints at different roles for the subunits in eIF2 assembly.

### eIF2α, but not eIF2β, directly associates with Cdc123

eIF2γ is the central protein in the eIF2 complex and directly binds eIF2α and eIF2β and also Cdc123. A conclusion as to whether the interactions between yeast eIF2α (Sui2) and eIF2β (Sui3) with Cdc123 are direct or mediated by yeast eIF2γ (Gcd11) could not be drawn from the previous experiment. Hence, we created variants of Sui2 and Sui3 with mutations in their Gcd11-binding sites. For both proteins, we chose conserved amino acids that were known to mediate the interaction with IF2γ in the archaeal ortholog (11, 25).

**Figure 4:**
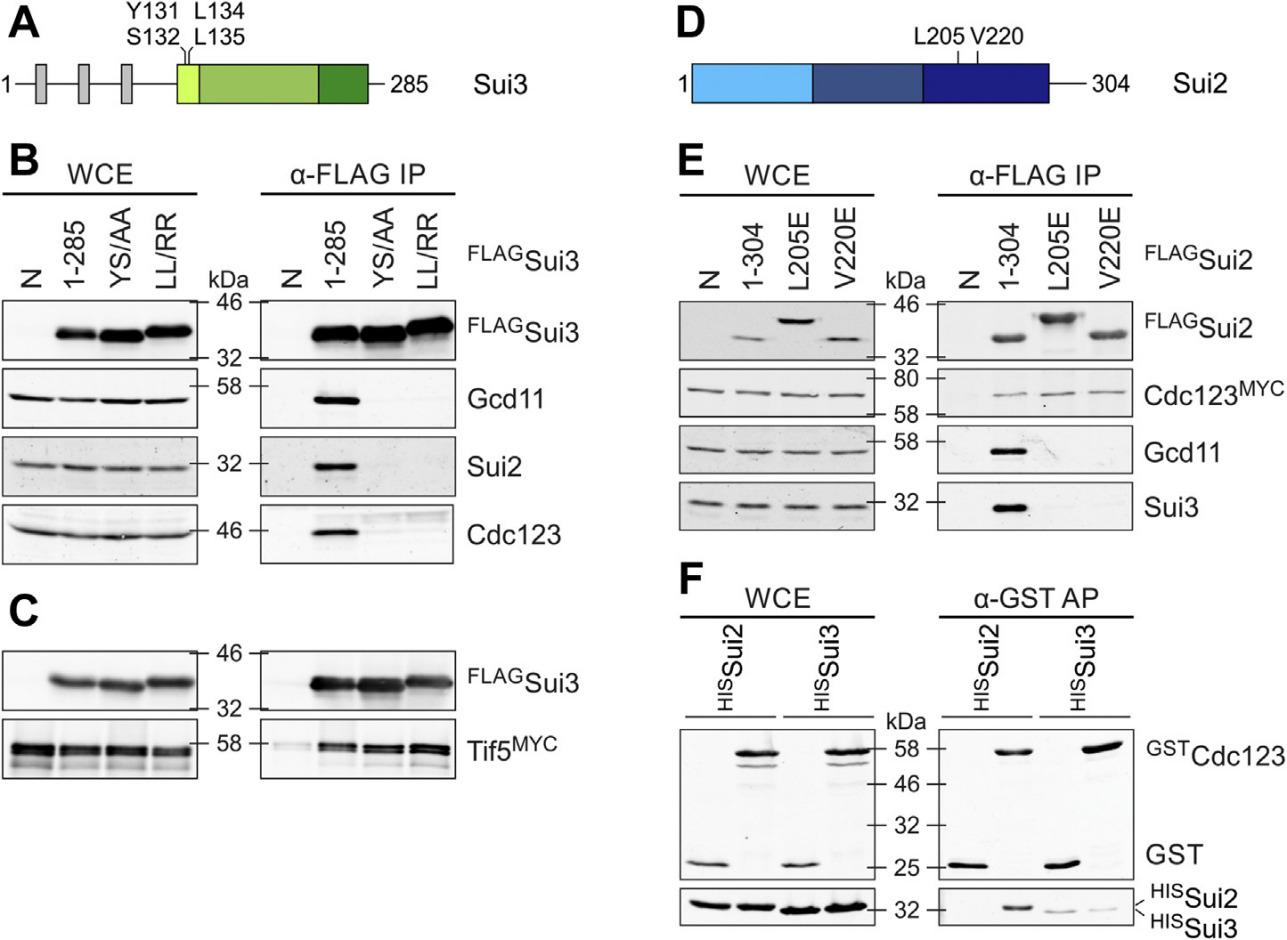
**Characterization of Cdc123–eIF2α and Cdc123–eIF2αβ interactions.***A*, schematic domain structure of eIF2β (Sui3). N-terminal lysine (K) boxes are shown in *gray*, domain I (amino acids 125–141) in *light green*, domain II (amino acids 142–233) in *green*, and domain III (amino acids 234–272) in *dark green* (33, 43). Numbers indicate amino acid positions of mutated versions of Sui3 used for IP (*B* and *C*). *B* and *C*, WB analysis of interactions between FLAG-tagged versions of Sui3 with eIF2γ (Gcd11), eIF2α (Sui2), Cdc123, and eIF5 (Tif5) in co-IPs. *D*, schematic domain structure of eIF2α (Sui2). Domain I (amino acids 1–93) is shown in *light blue*, domain II (amino acids 94–177) in *blue*, and domain III (amino acids 178–275) in *dark blue* (33, 43). Numbers indicate amino acid positions of mutated versions of Sui2 used for IP (*E*). *E*, interaction of FLAG-tagged versions of Sui2 with myc-tagged Cdc123, eIF2γ (Gcd11), and eIF2β (Sui3) was analyzed using IP. WCE and α-FLAG-immunoprecipitates were analyzed by WB. *F*, interaction of Cdc123 with eIF2α (Sui2) and eIF2β (Sui3). GST-Cdc123 and His-tagged eIF2α (^His^Sui2) or eIF2β (^His^Sui3) were coexpressed in *Escherichia coli*. *E. coli* cells expressing GST without Cdc123 were included as negative control. GST and GST-Cdc123 were affinity-purified on glutathione agarose beads. WCE and α-GST-affinity precipitates (APs) were analyzed by WB. In all analyses, protein levels were assessed in WCE (*left panels*) and after α-FLAG IP (*right panels*); analyses included no-tag controls (N). eIF2, eukaryotic translation initiation factor 2; GST, glutathione-*S*-transferase; IP, immunoprecipitation; WB, Western blot; WCE, whole cell extract.

First, we created two Sui3 mutants with double amino acid exchanges, Y131A/S132A and L134R/L135R (referred to as YS/AA and LL/RR; Fig. 4*A*) by site-directed mutagenesis. These amino acids are contained in the conserved domain I of eIF2β, and their archaeal counterparts are essential for binding aIF2γ (25). We expressed them with N-terminal FLAG tags, controlled by the repressible p*GAL1*, since Sui3 overexpression was found to compromise cell growth. Co-IPs, followed by WB, were performed, and binding of Sui2, Gcd11, and Cdc123 was analyzed. As predicted, neither mutant was able to bind Gcd11, confirming the high conservation of IF2 structures throughout the kingdoms of life. In addition, we detected no measurable amounts of Sui2, in accordance to established structural data (11, 25, 29). Importantly, Sui3 mutants defective in Gcd11 binding also failed to coprecipitate Cdc123 (Fig. 4*B*). We thus conclude that the interaction between Sui3 and Cdc123 is indirect and mediated by Gcd11. To address the possibility of structural defectiveness of Sui3 mutants, we tested interaction with yeast eIF5 (Tif5), a known interaction partner of Sui3 (30). We used strains in which Tif5 was C-terminally fused to a 13xMYC tag for detection. Co-IP revealed unaffected Sui3–Tif5 interaction for both mutants (Fig. 4*C*), validating our findings on Sui3–Cdc123 interaction.

Next, we created Sui2 mutants defective in Gcd11 binding. We chose the conserved amino acid positions L205 and V220 in domain III (Fig. 4*D*) and replaced these hydrophobic residues with acidic glutamate residues using site-directed mutagenesis. The variants were expressed with N-terminal 3xFLAG tags under control of the constitutively active *TEF2* promoter. The mutated Sui2 variants did not bind Sui3 or Gcd11 in the co-IP experiment, confirming the functional conservation of amino acid residues among eIF2α orthologs. The weak interaction with Cdc123 (Fig. 3, *E* and *F*), on the other hand, was not further reduced (Fig. 4*E*). While the result shows that interaction between Cdc123 and Sui2 is independent of Gcd11, we could not rule out the possibility of an unknown interaction partner bridging the two proteins. Hence, we wanted to verify the interaction in *Escherichia coli*, which does not have an eIF2 homolog or Cdc123. For this purpose, we expressed glutathione-*S*-transferase (GST)-tagged Cdc123 together with His-tagged Sui2 and Sui3 and pulled down Cdc123 with a GST affinity matrix. Coprecipitation of Sui2 and Sui3 was tested *via* WB, and we found a significant amount of Sui2 in the affinity precipitate eluate. Sui3 was not detected above background levels (Fig. 4*F*). We therefore conclude that Cdc123 establishes a direct contact to eIF2α but not to eIF2β. This contact is independent of Gcd11 and seems to be short lived, based on the low level of complexes containing Cdc123 and Sui2.

### eIF2α is rate limiting in eIF2 assembly

**Figure 5:**
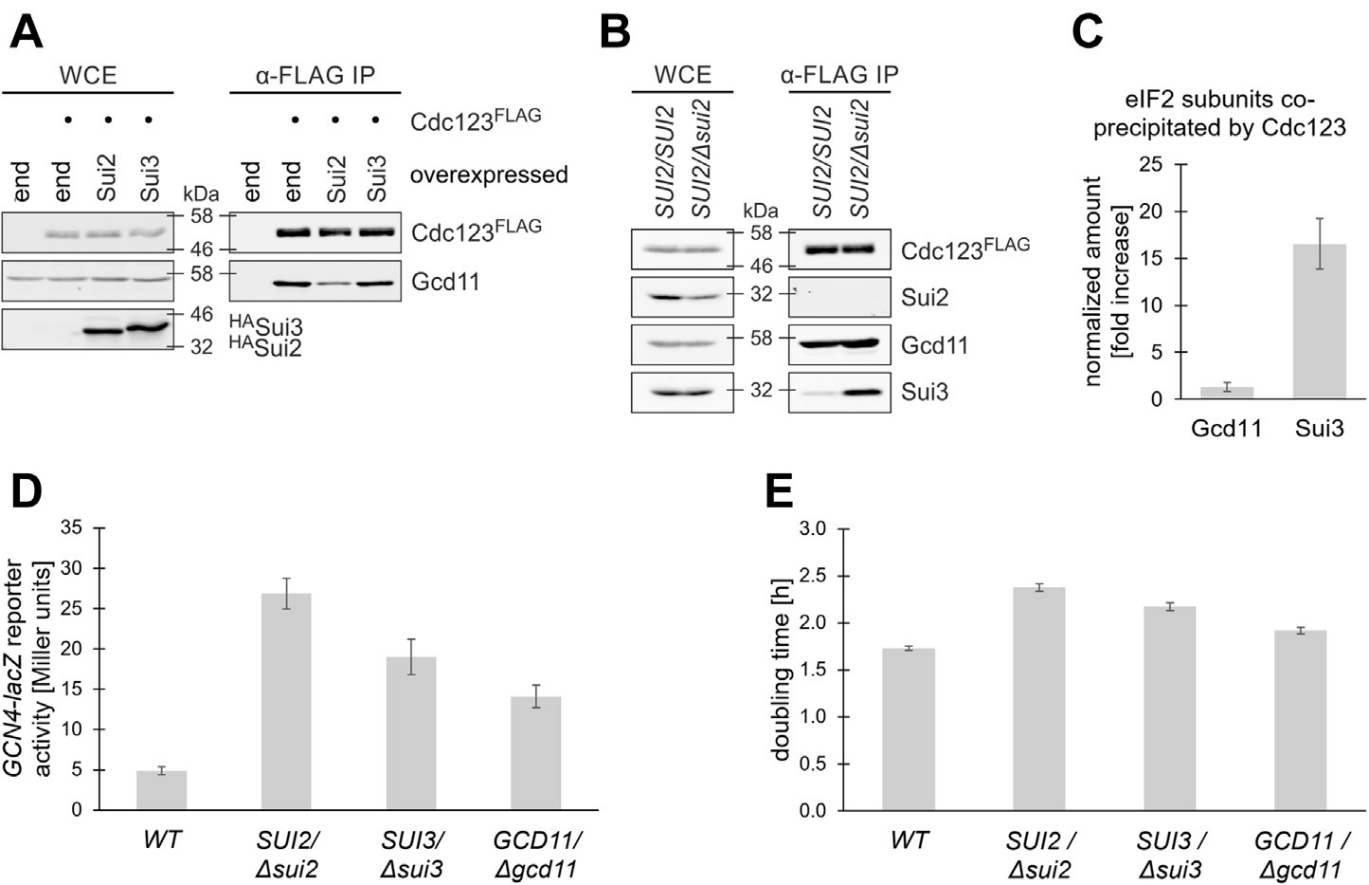
**Importance of eIF2α for eIF2 assembly and cell growth.***A*, WB analysis of interactions between Cdc123^FLAG^ with eIF2γ (Gcd11) in strains overexpressing either eIF2α (Sui2) or eIF2β (Sui3). The analysis included strains with endogenous levels of both proteins (end) and a no-tag control lacking Cdc123^FLAG^. *SUI2* and *SUI3* were expressed under control of the *GAL1* promoter. Protein levels were analyzed in WCE (*left panel*) and after α-FLAG IP (*right panel*). *B*, WB analysis of interactions between Cdc123^FLAG^ and Sui2, Sui3, and Gcd11 in a *SUI2*/*SUI2* homozygous and a *sui2Δ/SUI2* heterozygous diploid strain. *C*, quantification of coprecipitated Gcd11 and Sui3 in (*B*). Signals of immunoprecipitated Cdc123 and coprecipitated Gcd11 and Sui3 were quantified by use of an infrared imaging system. Signals of Gcd11 and Sui3 were normalized to the amount of the corresponding Cdc123^FLAG^ signal. The increase of coprecipitated eIF2 subunits in the *sui2Δ/SUI2* heterozygous diploid strain is shown as mean and SD (n = 4). *D*, *GCN4*-*lacZ* reporter levels were measured in a WT control and diploid strains heterozygous for the indicated eIF2 subunit gene deletion. β-Galactosidase activity in Miller units is shown as mean and SD (n = 6). *E*, doubling time of a WT control and the indicated heterozygous diploid strains. Doubling time is shown as mean and SD (n = 4). eIF2, eukaryotic translation initiation factor 2; IP, immunoprecipitation; WB, Western blot; WCE, whole cell extract.

After finding a direct but weak interaction between yeast eIF2α (Sui2) and Cdc123, we addressed the role of eIF2α in eIF2 assembly. To analyze the consequences of increased eIF2α levels, we overexpressed HA-tagged Sui2 or Sui3 in a strain with FLAG-tagged Cdc123. Cdc123^FLAG^ was pulled down by IP, and coprecipitated Gcd11 was detected by WB analysis. We found that the amount of copurified Gcd11 was markedly reduced in the Sui2-overexpressing strain (Fig. 5*A*). Since Gcd11–Cdc123 complexes are immature eIF2 assembly intermediates, this may indicate faster eIF2 assembly. We hypothesize that eIF2α might sterically clash with Cdc123 on the eIF2γ platform and facilitate release of Cdc123. Since Sui2 overexpression can apparently speed up eIF2 assembly, we asked if lowering its abundance may slow down assembly and lead to an increase in eIF2 assembly intermediates. To this end, we introduced Cdc123^FLAG^ constructs into a diploid yeast strain with only one *SUI2* gene copy. We expected Sui2 expression in this strain to be reduced to around 50% compared with WT. IP of Cdc123^FLAG^ was performed, and interaction partners of Cdc123 were quantified by WB analysis. As expected, the Sui2 protein level was reduced to ∼50% in the heterozygous diploid strain, and coprecipitation of Sui3 with Cdc123^FLAG^ was increased more than 15-fold (Fig. 5, *B* and *C*). The elevated level of this assembly intermediate argues that eIF2 assembly is delayed when eIF2α levels are lowered. Coprecipitation of Gcd11 was only moderately increased by 1.3-fold. This is possibly explained by our previous findings that unassembled Gcd11 is mostly present in heterodimeric complexes with Cdc123 and to a lesser degree in various eIF2 assembly intermediates. Sui3 on the other hand does not form dimeric complexes with Cdc123 (Fig. 4*B*). Its association with Cdc123 is limited to trimeric complexes with Gcd11 and Cdc123, which are short lived because Cdc123 is quickly released after Sui2 binding. Together, the data indicate that the eIF2γ–Cdc123 association dissolves in response to elevated eIF2α levels, whereas an eIF2γ–eIF2β–Cdc123 assembly intermediate accumulates when eIF2α levels are lowered.

To further define the consequences of low eIF2α levels, we compared eIF2 functionality and cell growth in diploid yeast strains carrying heterozygous deletions of eIF2 subunit genes. To this end, we measured *GCN4* mRNA translation using a well-established reporter construct containing the *GCN4* 5′-leader in front of the *lacZ* gene (31). Indeed, the heterozygous deletion of *SUI2* increased the *GCN4*-*lacZ* activity more than deletions of either *SUI3* or *GCD11* did (Fig. 5*D*; *sui2Δ/SUI2*: 27 ± 2 Miller units; *sui3Δ/SUI3*: 19 ± 2; *gcd11Δ/GCD11*: 14 ± 1; and WT: 5 ± 0.5; n = 6). We measured cell proliferation during exponential growth. Again, heterozygous deletion of *SUI2* caused the strongest effect (Fig. 5*E*; *sui2Δ/SUI2*: 2.38 ± 0.04 h; *sui3Δ/SUI3*: 2.17 ± 0.04 h; *gcd11Δ/GCD11*: 1.92 ± 0.04 h; and WT: 1.74 ± 0.00 h; n = 4). Growth deficiency thus correlated well with eIF2 function. Together, our data suggest that eIF2α abundance is rate limiting in eIF2 assembly. While all subunits are essential parts of eIF2, eIF2α may in addition fulfill an active role in eIF2 complex formation.

### eIF2α-γ interaction is required for Cdc123 release

**Figure 6:**
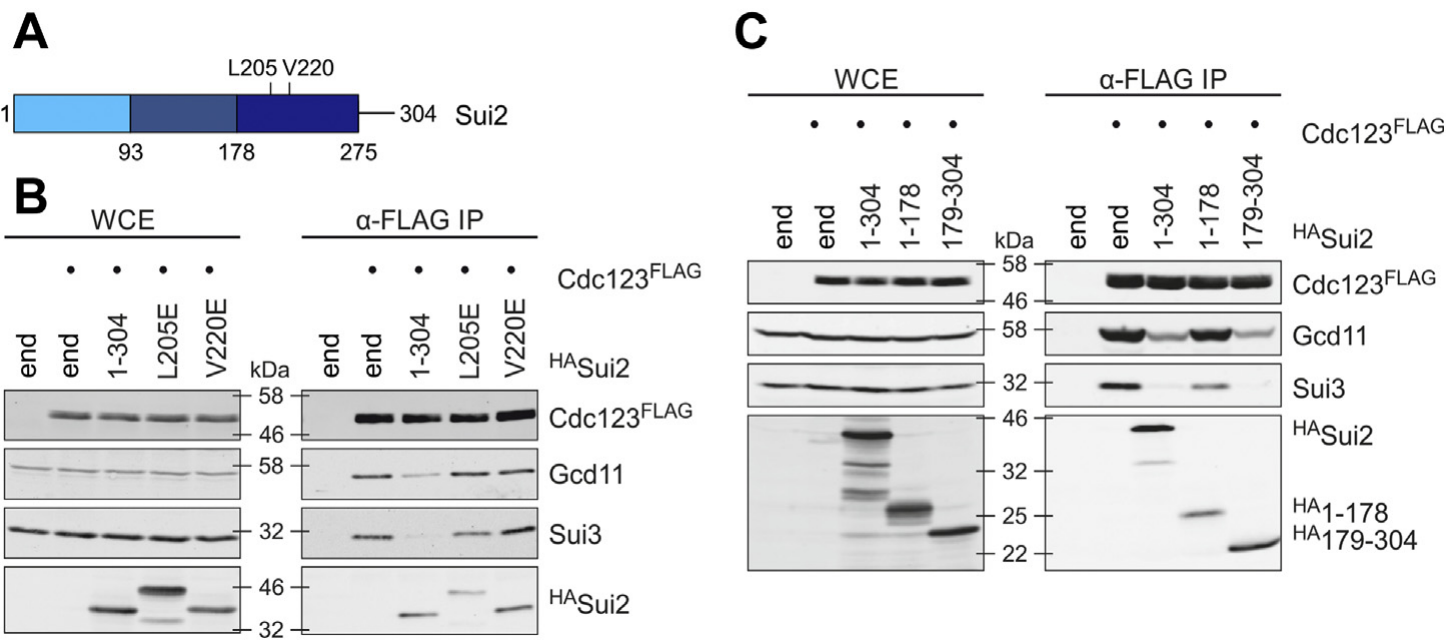
**eIF2α-mediated Cdc123 release requires binding of eIF2α to eIF2γ.***A*, schematic domain structure of eIF2α (Sui2). *B* and *C*, WB analysis of interactions between Cdc123^FLAG^ with eIF2β (Sui3) and Gcd11 (eIF2γ) in strains overexpressing variants of eIF2α (^HA^Sui2). The analyses included strains with endogenous Sui2 levels (end). Strains lacking Cdc123^FLAG^ served as no-tag controls (*left lane* in all panels). HA-tagged *SUI2* variants were expressed under control of the *GAL1* promotor. eIF2, eukaryotic translation initiation factor 2; HA, hemagglutinin; WB, Western blot.

Our observations suggest that eIF2α may act as a release factor for Cdc123 to complete eIF2 assembly. However, details about the mechanism remain unclear, specifically whether the release is catalyzed solely *via* an interaction between eIF2α and Cdc123 or whether eIF2α must bind eIF2γ for the release to take place. To address this question, we investigated the capability of Sui2 variants to dissolve Gcd11–Cdc123 complexes. First, we overexpressed Sui2 variants L205E and V220E, which are incapable of binding Gcd11 (Fig. 4, *D* and *E*) or Sui2-WT in yeast strains with FLAG-tagged Cdc123. Again, we precipitated Cdc123^FLAG^ and analyzed coprecipitation of eIF2 subunits *via* WB. Consistent with our previous results (Fig. 5*A*), we observed a reduction in Cdc123^FLAG^–Gcd11 and Cdc123^FLAG^–Sui3 interactions in strains overexpressing WT Sui2. The variants L205E and V220E, however, had no significant effect on either interaction (Fig. 6*B*). Next, we created a C-terminally truncated Sui2 variant (amino acids 1–178) as well as an N-terminally truncated one (179–304) and used them in the same setup. Based on structural data of human eIF2α, the protein consists of two parts, domain I + II and domain III, which are mobile relative to each other (32). Crystal structures of archaeal IF2 suggest that domain III of IF2α, which in yeast starts at amino acid 179, contains all contact points to IF2γ, including L205 and V220 (Fig. 6*A*) (11). In accordance with our expectations, WT Sui2 and Sui2 179 to 304 reduced the amount of Cdc123–Gcd11 and Cdc123–Sui3 complexes. The C-terminally truncated variant had little or no effect on Gcd11 coprecipitation and a minor one on Sui3 coprecipitation. Both Sui2 fragments were coprecipitated by Cdc123^FLAG^ (Fig. 6*C*). Our data thus suggest that the eIF2α-mediated release of Cdc123 takes place on the eIF2γ platform and may involve direct contacts between eIF2α and Cdc123.

### Cdc123 dissolves intramolecular interactions between eIF2γ domains

**Figure 7:**
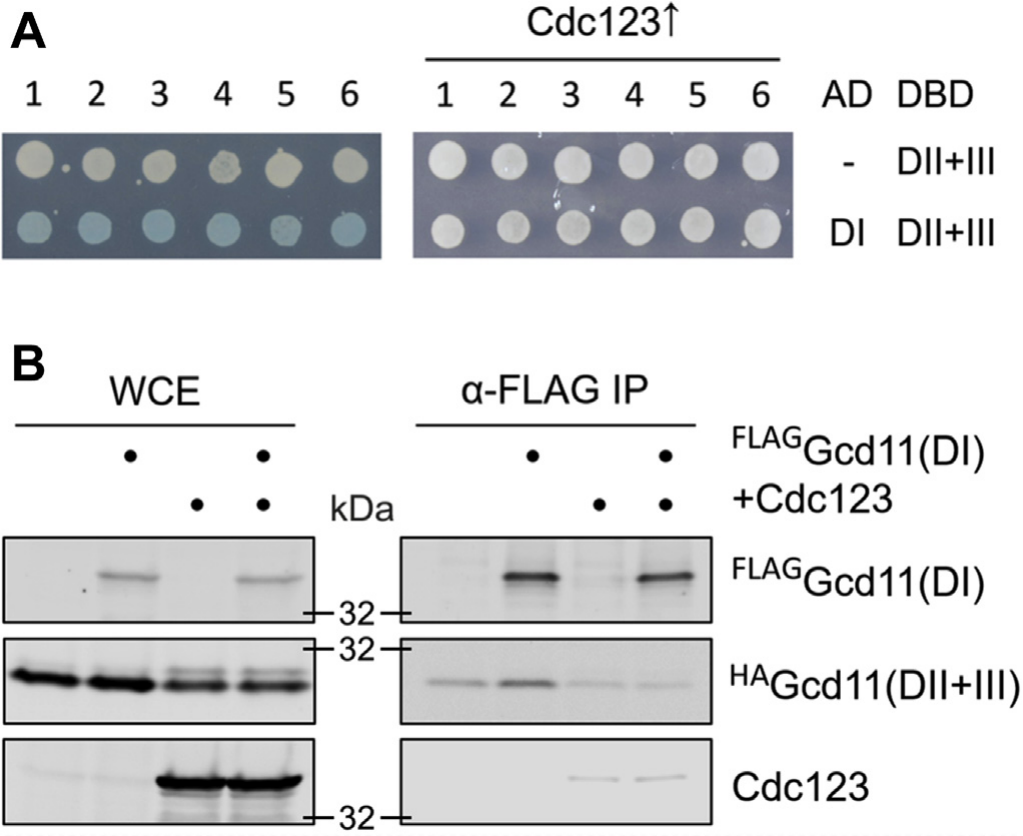
**Intramolecular interaction between Gcd11 domains dissolved by Cdc123.***A*, interaction between Gcd11 domain I and domain II + III fragments was analyzed in a Y2H assay using fragment DI fused to the activator domain (AD) and fragment DII + III fused to the LexA DNA-binding domain (DBD). The experiment was performed in a regular reporter strain with endogenous Cdc123 levels (*left panel*) and a modified reporter strain overexpressing Cdc123 (*right panel*). For each combination, six independent transformants are shown. *Blue color* indicates activation of the *lacZ* reporter gene. *B*, WB analysis of interaction between DI and DII + III fragments of Gcd11. Protein levels were analyzed in WCE (*left panel*) and after α-FLAG IP (*right panel*). The analysis included no-tag controls lacking ^FLAG^Gcd11(DI). The interaction was analyzed in yeast strains with natural and increased levels of Cdc123 expression. IP, immunoprecipitation; WB, Western blot; WCE, whole cell extract; Y2H, yeast two-hybrid assay.

Cdc123 is sometimes referred to as an eIF2γ chaperone (24), owing to its putative role in altering eIF2γ structure. Some previous observations, for example, the loss of interaction with Sui2 in the Gcd11 (amino acids 91–527) variant, hint at the possibility of interdomain communication within eIF2γ. In addition, domain I of IF2γ was seen to contact domain 3 in archaea (12). To test, whether interdomain interaction occurs in Gcd11, we tested the binding of Gcd11 domain I and domain II + III fragments in a yeast two-hybrid (Y2H) assay. The N-terminal fragment was tagged with the transcriptional activator domain, whereas the C-terminal fragment was fused to the LexA DNA-binding domain. Indeed, a moderate interaction between domain I and domain II + III fragments was observed, as shown by the visible β-galactosidase activity (Fig. 7*A*, *left panel*). Next, we repeated the experiment in a modified reporter strain, which overexpressed Cdc123. This time, no interdomain binding was observed (Fig. 7*A*, *right panel*). This could mean that Cdc123 introduces a change in Gcd11 that alters the way in which interdomain communication takes place. To substantiate this finding, we then investigated the interaction in co-IPs. The domain I fragment was FLAG tagged, and the domain II + III fragment coupled to an HA tag for detection. We carried out the IP in a strain with endogenous levels of Cdc123 and a second one that overexpressed this assembly factor. A low amount of ^HA^Gcd11(DII + III) was coprecipitated by ^FLAG^Gcd11(DI) in the strain with endogenous Cdc123 levels, but overexpression of Cdc123 broke up the interaction. ^FLAG^Gcd11(DI) did not coprecipitate Cdc123 at above-background levels (Fig. 7*B*). We concluded that Cdc123 may alter the structure of eIF2γ in a way that modulates its interdomain communication. Associations between domain I and domain II + III may be a property of Cdc123-naïve eIF2γ.

### Similarity of eIF2 assembly in yeast and humans

**Figure 8:**
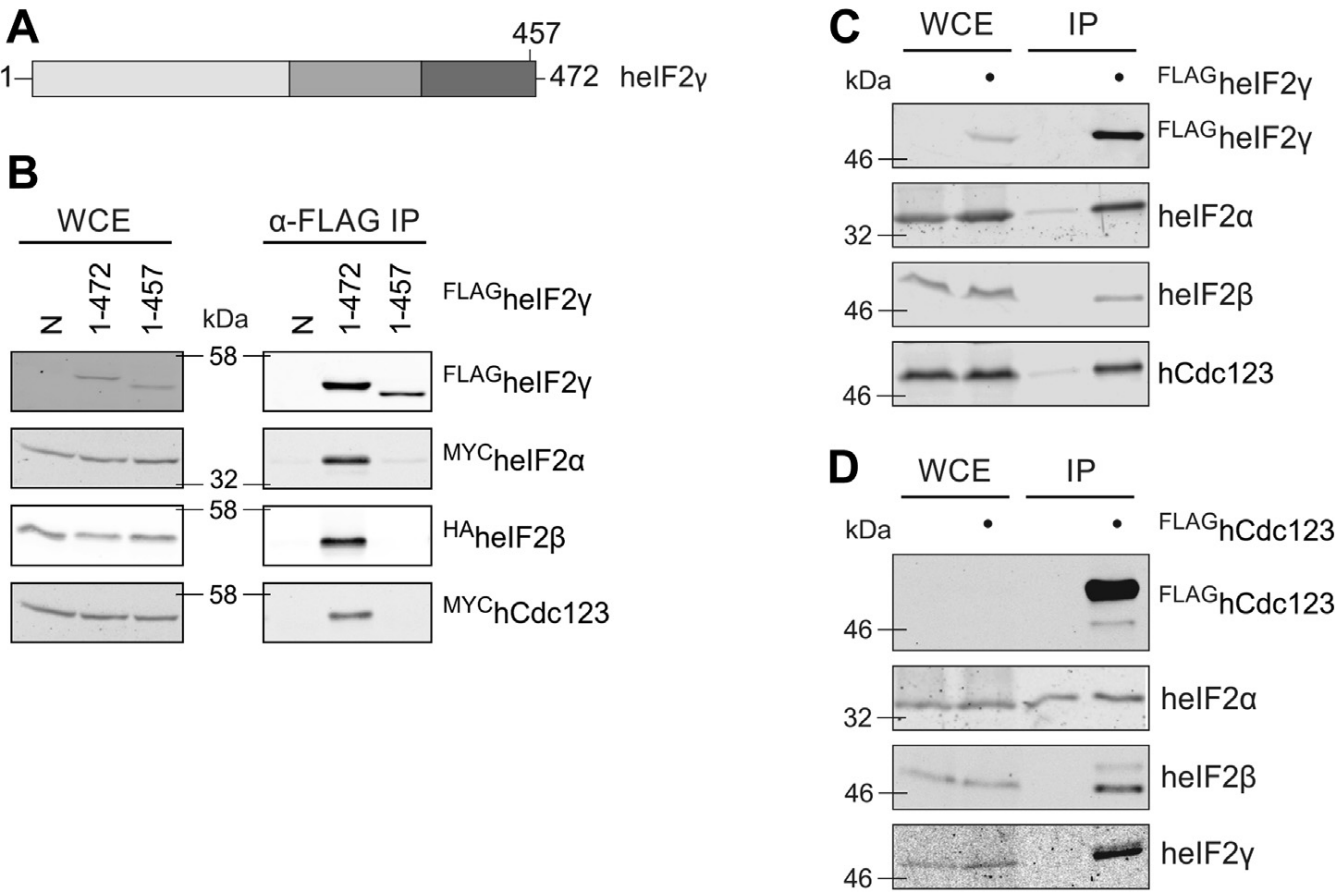
**Interactions between human eIF2 subunits and human Cdc123.***A*, schematic domain structure of human eIF2γ. Domain I (G-domain) is shown in *light gray*, domain II in *gray*, and domain III in *dark gray*. *B*, WB analysis of interactions between human ^FLAG^eIF2γ with human ^MYC^eIF2α, ^HA^eIF2β, and ^MYC^Cdc123 proteins in yeast cells. Protein levels were analyzed in WCE and after α-FLAG IP. No tag controls (N) were included. *C*, WB analysis of interaction between ^FLAG^heIF2γ with heIF2α, heIF2β, and hCdc123 in Flp-In T-REx-293 cells. *D*, WB analysis of interactions between ^FLAG^hCdc123 with heIF2α, heIF2β, and heIF2γ in Flp-In T-REx-293 cells. *C* and *D*, protein levels were analyzed in WCE and after α-FLAG IP, and no-tag controls were included. eIF2, eukaryotic translation initiation factor 2; IP, immunoprecipitation; WB, Western blot; WCE, whole cell extract.

eIF2 function and translation initiation in general are highly conserved among eukaryotes (1, 2). Moreover, human Cdc123 can rescue yeast cells deprived of endogenous Cdc123 (17, 21). It was therefore of interest to see whether, in case of the human proteins, integrity of the eIF2γ C terminus is required for the eIF2γ–Cdc123 interaction, and for eIF2 assembly, as was previously described for yeast (14). For this, we created yeast strains that express human (h) ^MYC^eIF2α, ^HA^eIF2β, and ^MYC^Cdc123 together with full-length ^FLAG^heIF2γ or a truncated version of the protein (amino acids 1–457; Fig. 8*A*). FLAG-IPs were performed, and coprecipitation of heIF2 subunits and hCdc123 was investigated. We observed a robust interaction between full-length heIF2γ with its putative interaction partners. Similar to the situation in yeast, the C-terminally truncated variant of heIF2γ lacked interaction with hCdc123 and failed to bind heIF2α and heIF2β (Fig. 8*B*). This supports the view that the pathways of eIF2 complex formation are similar in yeast and humans.

To study eIF2 assembly in human cells, we introduced single copies of human ^FLAG^eIF2γ and ^FLAG^Cdc123 into a human embryonic kidney–derived cell line. We precipitated ^FLAG^heIF2γ and ^FLAG^hCdc123 and analyzed associated proteins *via* WB. As expected, we detected signals for heIF2α, heIF2-β, and hCdc123 in the ^FLAG^heIF2γ-IP (Fig. 8*C*). Likewise, ^FLAG^hCdc123 precipitated significant amounts of heIF2γ and moderate amounts of heIF2β, whereas the heIF2α signal was barely above background. When comparing the signals for each protein between whole cell extract and IP, heIF2γ was enriched the most and heIF2α the least, with heIF2β showing intermediate enrichment (Fig. 8*D*; heIF2γ: 100%, heIF2β: 37%, and heIF2α: 3.4%, n = 1). These results indicate that Cdc123–eIF2 protein complexes occur in similar proportions in human and yeast cells. This points to conservation of the basic mechanism of eIF2 assembly.

## Discussion

**Figure 9:**
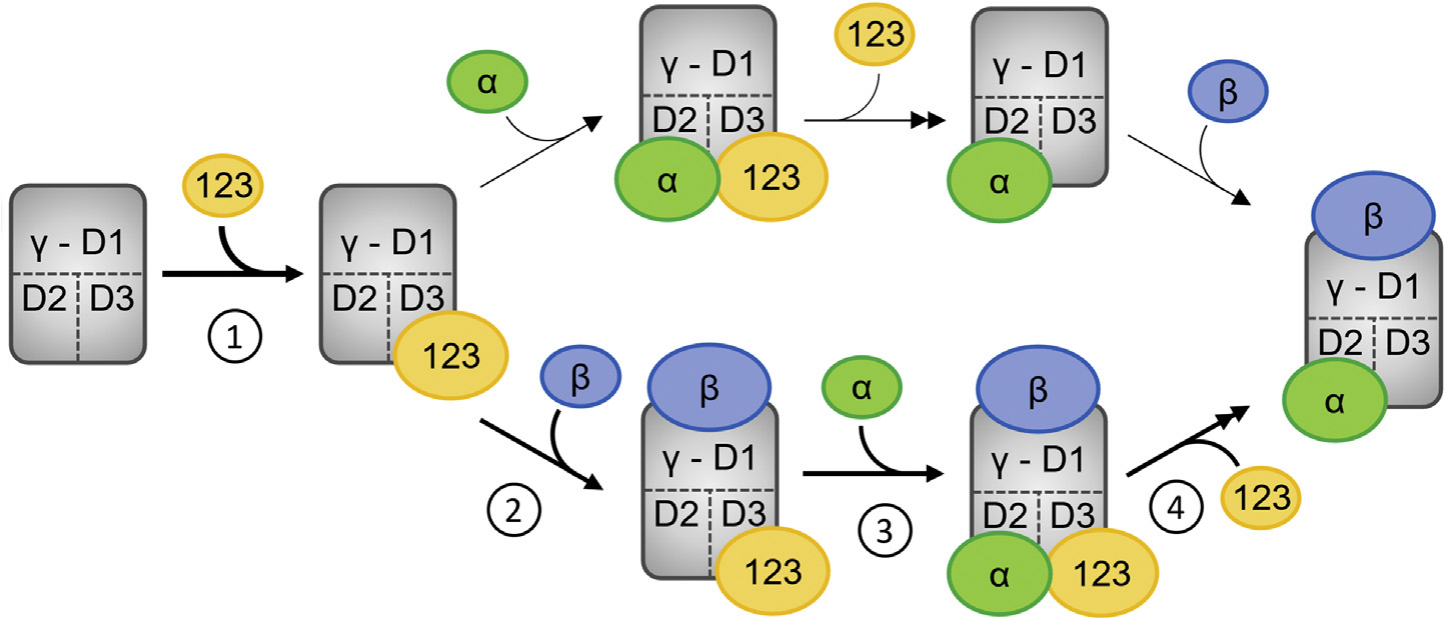
**Model of eIF2 assembly.** Numbers indicate the distinct steps of the assembly pathway. (1) Cdc123 associates with the C-terminal part of eIF2γ and activates the eIF2α and eIF2β binding sites, possibly *via* structural change of eIF2γ. This enables eIF2α and eIF2β to bind. (2) eIF2β binds and a detectable eIF2γβ–Cdc123 trimer is formed. (3) eIF2α binds the assembly intermediate and destabilizes the Cdc123–eIF2γ interaction. Therefore, the tetrameric eIF2αγβ–Cdc123 intermediate is a transient structure, and its abundance *in vivo* is very low. (4) Cdc123 quickly dissociates (as indicated by the *double arrow*) and leaves behind the trimeric eIF2 complex. *Upper part*, in a putative secondary reaction pathway, eIF2α, rather than eIF2β, binds the eIF2γ–Cdc123 dimer. Cdc123 dissociates and leaves behind an eIF2γα dimer, to which eIF2β binds. The final product of both reaction pathways is trimeric eIF2. eIF2, eukaryotic translation initiation factor 2.

eIF2 is a central player in translation initiation. Its mechanistic function in initiator-tRNA recruitment and start codon recognition as well as its role in the regulation of stress-induced gene expression has been studied in considerable detail (1, 2, 7, 8, 33, 34, 35). Relatively little, however, is known about the initial formation of the heterotrimeric eIF2 protein complex, even though this process is essential for translation initiation and cell viability (14). Here, we studied the assembly of eIF2 in *S. cerevisiae* in detail. This included the quantification of assembly intermediates and the use of binding site mutants of eIF2 subunits to analyze individual interactions between eIF2α, eIF2β, eIF2γ, and Cdc123. Together, the data allow us to propose a model for the stepwise assembly of eIF2 (Fig. 9). Since assembly intermediates were detected in similar quantities in human cells (Fig. 8*D*), the proposed model may apply also to other eukaryotic organisms.

In the first step of the assembly model, Cdc123 binds to domain III of newly synthesized eIF2γ, and this association is a prerequisite for the subsequent binding of the α-subunit and the β-subunit to eIF2γ (14) (Fig. 2*B*). The precise Cdc123–eIF2γ binding interface has been resolved by X-ray structural data (19). Cdc123 belongs to the family of ATP grasp proteins, whose members typically catalyze carboxyl-amino linkages and may thereby introduce post-translational modifications into target proteins (20). But so far, no such Cdc123-mediated modification of eIF2γ has been detected. However, we found that Cdc123 can interrupt the intramolecular association of the G-domain of eIF2γ with domains II + III (Fig. 7). This association could be a feature of immature Cdc123-naïve eIF2γ and prevent the α-subunit and the β-subunit from binding, possibly by hiding important interaction surfaces. Cdc123 might serve as an allosteric activator and introduce a structural change in eIF2γ to open up those interaction surfaces. The assumed interdomain communication in eIF2γ is supported by our finding that removal of amino acids 1 to 90 in yeast eIF2γ affects eIF2α and eIF2β binding equally (Fig. 1), even though eIF2α binds exclusively to eIF2γ-DII (6, 33, 36).

The analysis of binding site mutants of eIF2γ indicated that eIF2α and eIF2β are largely independent of each other in binding to eIF2γ (Fig. 2*B*). However, a trimeric Cdc123–eIF2γ–eIF2β intermediate was present in cell extracts at much higher levels than any complex containing both Cdc123 and eIF2α (Fig. 3, *C*–*F*). This provides evidence for an assembly pathway in which eIF2β associates with the Cdc123-bound eIF2γ subunit before eIF2α joins in (Fig. 9, *lower part*). Indeed, eIF2α binding to eIF2γ appears to displace Cdc123 and thereby complete assembly of the eIF2 complex. The notion of eIF2α acting to release the Cdc123 assembly factor derives from the finding that eIF2α, when overexpressed, reduced the binding of Cdc123 to eIF2γ, whereas eIF2β failed to do so (Fig. 5*A*). The Cdc123-release activity of eIF2α might actually be rate limiting for eIF2 complex formation *in vivo*, since reduced amounts of eIF2α resulted in elevated levels of the trimeric Cdc123–eIF2γ–eIF2β assembly intermediate (Fig. 5, *B* and *C*). Moreover, reduced amounts of eIF2α decreased the functionality of eIF2 and overall cell growth to a greater extent than equal reductions of eIF2β or eIF2γ (Fig. 5, *D* and *E*). To release Cdc123 from eIF2γ, eIF2α needs to bind eIF2γ (Fig. 6) even though eIF2α can contact Cdc123 directly (Fig. 4, *E* and *F*). Thus, the release of Cdc123 may involve both an allosteric effect by which association of eIF2α with its binding site in eIF2γ-DII alters the remote Cdc123-binding site in eIF2γ-DIII as well as a physical displacement through direct contact. This perception is consistent with previous structural modeling data (19). Further support for the role of eIF2α in liberating the assembly factor comes from the finding that an eIF2γ variant defective in eIF2α binding can trap Cdc123 and interfere with cell growth (Figs. 2*B* and S1). These assays also suggested that eIF2β binding to eIF2γ may contribute to some degree to the release of the assembly factor.

The assembly pathway proposed in this study (Fig. 9, *lower part*) is based on the detection of the Cdc123–eIF2γ–eIF2β intermediate (Fig. 3). The data, however, do not rule out an additional route in which the binding of eIF2α to Cdc123-activated eIF2γ precedes the association of eIF2β (Fig. 9, *upper part*). The predicted Cdc123–eIF2γ–eIF2α intermediate would have to be very short lived, since complexes containing Cdc123 and eIF2α are rare (Fig. 3). This route would also require that the eIF2γ–eIF2α dimer remains in a state competent for eIF2β binding after Cdc123 dissociation. Future studies on the detailed mechanism of action of Cdc123 may resolve this aspect.

After completion of its assembly, the heterotrimeric eIF2 complex can bind the GEF eIF2B, which promotes GTP binding to eIF2. Our data indicate that the dimeric assembly intermediates eIF2γ–eIF2α and eIF2γ–eIF2β do not support cell viability (Fig. 2*E*) and also fail to associate with the GEF eIF2B (Fig. 2, *C* and *D*). This is in line with structural and protein interaction data, in which all three eIF2 subunits were found to contact eIF2B (29, 37). After guanosine nucleotide exchange, the initiator-tRNA binds to eIF2-GTP whereby a functional TC is formed (1). The TC then binds to the 40S ribosomal subunit and enters the cycle of translation initiation.

The significance of proper assembly of the eIF2 complex is demonstrated also by disease mutations of human eIF2γ (22, 24). A recently described frame-shift mutation (eIF2γ-I465fs∗4) alters the most C-terminal portion of human eIF2γ and gives rise to a severe neurological disease syndrome (24). This mutation was reported to impair the Cdc123-promoted assembly of eIF2γ with eIF2α. Consistent with this work, we observed that small C-terminal truncations of yeast and human eIF2γ impede their association with the Cdc123 assembly factor from the respective organism and consequently also formation of the eIF2 complex (Figs. 2*B* and 8*B*). Moreover, an assembly intermediate of Cdc123 with eIF2γ and eIF2β was detected in comparable quantities in yeast and human cells (Figs. 3, *C*–*F* and 8*D*) arguing that eIF2 assembly may take similar steps in distinct organisms. Thus, the details of eIF2 assembly throw light on a conserved process essential for protein synthesis in eukaryotes.

## Experimental procedures

### Yeast methods

We followed standard protocols for yeast cultivation, transformation, crossing, sporulation and tetrad dissection, and Y2H analysis (38). All strains are derivatives of W303 and listed in Table S2. Yeast cells were grown in XY medium, which is based on yeast extract–peptone–dextrose medium and supplemented with 100 mg/l adenine, 200 mg/l tryptophan, and 10 mM KH_2_PO_4_. Carbon sources, glucose (D), raffinose (R), or galactose (G), were added in a concentration of 2%. Generally, liquid cultures were incubated at 25 °C. Cell lysates were extracted from exponentially growing liquid overnight cultures. For spot growth assays, exponentially growing cultures were harvested and resuspended in water at an absorbance of 1 at 600 nm. Cells were serially diluted 10-fold and spotted on agar plates. Inoculated agar plates were incubated at 22 to 30 °C for 2 to 4 days. For liquid culture growth assays, a defined amount of cells from exponentially growing overnight cultures was transferred to multiple vials containing XY-D media. Absorbance at 600 nm values before and after cultivation was compared, and doubling time was calculated based on the duration of cultivation.

### LacZ reporter gene assay

*GCN4* expression was tested with a reporter plasmid, in which the *GCN4* 5′-leader was fused to the *lacZ* gene (31) (Table S1). Yeast strains were transformed with the reporter plasmid. Selected colonies were cultivated, and 1 ml of exponentially growing cells at an absorbance of 1 at 600 nm was harvested and resuspended in Z-buffer (60 mM Na_2_HPO_4_, 40 mM NaH_2_PO_4_, 10 mM KCl, 1 mM MgSO_4_, and 50 mM β-mercaptoethanol, pH = 7). 0.0025% SDS and 50 μl chloroform were added. Cells were shaken at 37 °C for 15 min. Then, 200 μl of a 0.4% *ortho*-nitrophenyl-*β*-galactoside solution was added and incubated with the cells for an appropriate amount of time. About 500 μl of a 1 M Na_2_CO_3_ solution was added to stop the reaction. Absorbance values at 420 nm were measured, and a cell-free sample was used as blank. Miller units were calculated as described (38).

### Molecular cloning

Yeast and human genes were amplified from pre-existing plasmids. Originally, yeast genes were amplified from yeast genomic DNA (BY4741) with primers containing appropriate restriction sites for molecular cloning. The human *EIF2S1-3* genes were obtained from DNASU Plasmid Repository (Arizona State University). Human *CDC123* was provided by C. Höög (39). PCR-amplified constructs were routinely sequenced (sequencing done by Microsynth Seqlab and GATC Biotech). Expression vectors for yeast were based on the pRS vector series (40). For heterologous gene expression in *E. coli*, vectors pJOE2955 and pJOE4056 were used. Plasmids are listed in Table S1.

### Genetic manipulation of yeast

For PCR-based C-terminal epitope taggings, plasmids from the pFA6a series were used (41). N-terminal tagging of endogenous genes was achieved by homologous recombination with plasmid fragments.

### Yeast cell lysis and IP

Cell lysis was performed as described by Schwab *et al.* (42). For IP, equal amounts of total cell protein (around 2 mg per sample) were incubated with 30 μl αFLAG affinity beads (Bimake) at 4 °C for 2 h. Beads were washed three times with lysis buffer, and proteins were eluted with 1× Laemmli sample buffer at 100 °C for 10 min.

### Mammalian cell cultivation, lysis, and IP

Mammalian cell lines are derivatives of Flp-In T-REx-293 cells (Thermo Fisher Scientific) and listed in Table S3. Standard Flp-In T-REx-293 cells were cultivated in Dulbecco's modified Eagle's medium + 10% fetal bovine serum (Thermo Fisher Scientific) with 10 μg/ml blasticidin and 100 μg/ml zeocin (Invivogen). Cells were passaged by trypsination one to two times per week. Stable cell lines were created according to protocols by Thermo Fisher Scientific for pcDNA5-FRT/TO and Flp-In T-REx-293. Plasmids used in mammalian cell culture are listed in Table S1. New cell lines were tested for protein expression by tetracyclin induction, followed by lysis and WB. We used 500,000 to 2 million cells for test expressions and around 100 million cells for IP. Cells were lysed by shaking in cell lysis buffer (150 mM NaCl, 50 mM Tris–HCl, pH = 8.2, 1% Triton X-100, 5 mM EDTA, 5 mM NaF, 0.2 mM 4-(2-aminoethyl)benzenesulfonyl fluoride hydrochloride, 2 μg/ml aprotinin, 2.5 μg/ml pepstatin; inhibitors added freshly before use) for 15 min. Equal amounts of lysate (around 8 mg per sample) were incubated with M2 antibody α-FLAG affinity matrix (Sigma–Aldrich) for 2 h at 4 °C. Elution was performed twice by incubation with 7.5 μg 3× FLAG peptide (Sigma–Aldrich) in 40 μl lysis buffer under vigorous shaking. Eluates were mixed with 4× Laemmli sample buffer (1× final concentration) and boiled for 5 min.

### WB analysis

SDS-PAGE and WB analysis were performed as described by Schwab *et al.* (42). Antibodies and antisera are listed in Tables S4 and S5. Antisera to Sui2, Sui3, Gcd11, and Sc-Cdc123 have been described by Perzlmaier *et al.* (14). Epitope-specific and fusion protein–specific antibodies and antibodies to human eIF2 subunits were obtained from commercial sources (Table S4). Rabbit antiserum to hCdc123 was produced by Davids Biotechnologie, using *E. coli*-expressed and affinity-purified His6-hCdc123 (amino acids 1–290) as an antigen. The serum was absorbed to nitrocellulose-bound his6-hCdc123 (amino acids 1–290) protein and eluted with 0.2 M glycine (pH = 2). The purified serum was immediately neutralized with 2 M Tris (pH = 7.5). All secondary antibodies are IRDye coupled and detected using the Odyssey Infrared Imaging System (LiCOR). LiCOR Odyssey, version 3.0 software was used for analysis and quantification of bands. For the quantification of protein bands in IP, signals in negative control samples were used as blanks and subtracted from the signals to be quantified.

### Protein expression in *E. coli* and affinity precipitation

*E. coli* strain BL21C+ was transformed with expression plasmids for His6-Sui2 or His6-Sui3 fusion proteins and GST or GST-Cdc123 proteins (Table S1). Transformants were cultivated overnight in liquid LB medium with 100 μg/ml ampicillin, 34 μg/ml chloramphenicol, and 50 μg/ml kanamycin. In the morning, new cultures were inoculated at an absorbance of 0.1 at 600 nm and grown at 25 °C for 2 h. Then, 0.2% rhamnose was added to induce protein expression, and cells were grown for another 20 h. Cell harvest, lysis, affinity precipitation, and WB analysis were performed analogous to yeast cell lysis and IP. Instead of 30 μl αFLAG agarose beads, 40 μl glutathione agarose beads (Thermo Fisher Scientific) were used, and incubation with lysates was performed for 3 h rather than 2 h.

### Y2H

Yeast strain W276 and its derivative W15023 were used as reporter strains. The reporter strain was transformed with two plasmids, based on pEG202 and pJG4-5 (38). An activator domain fusion of Gcd11(DI) was constructed in pJG4-5, and a DNA-binding domain fusion of Gcd11(DII + III) was created in pEG202 (Table S1). Six transformants were spotted on XYG-HT agar plates and incubated for 3 days at 25 °C. Then, a reaction solution (0.8 M sodium phosphate buffer [pH = 7.0], 10% dimethylformamide, 0.15% SDS, and 3 mg X-Gal) was mixed with 5 ml warm liquid agar and poured over the plate to cover the yeast cells. The plate was incubated at 30 °C for 24 h.

### Statistics

All statistics were calculated in Microsoft Office Excel.

## Data availability

All data needed for the understanding of this work are contained within the article.

## Supporting information

This article contains supporting information .

## Conflict of interest

The authors declare that they have no conflicts of interest with the contents of this article.
