# Supplementary material for: Stepwise assembly of the eukaryotic translation initiation factor 2 complex
Source: J Biol Chem. 2022 Jan 12;298(2):101583. doi: 10.1016/j.jbc.2022.101583 (PMC8844851; doi:10.1016/j.jbc.2022.101583)
Supplement: Supplemental Figures S1–S2 and Tables S1–S5 [file mmc1.docx]

**Supporting information**

Stepwise assembly of the eukaryotic translation initiation factor 2 complex

Sven Vanselow^1^, Lea Neumann-Arnold^1^, Franziska Wojciech-Moock^1^ and Wolfgang Seufert^1^

^1^Department of Genetics, Regensburg Center for Biochemistry,
University of Regensburg, D-93040 Regensburg, Germany

| 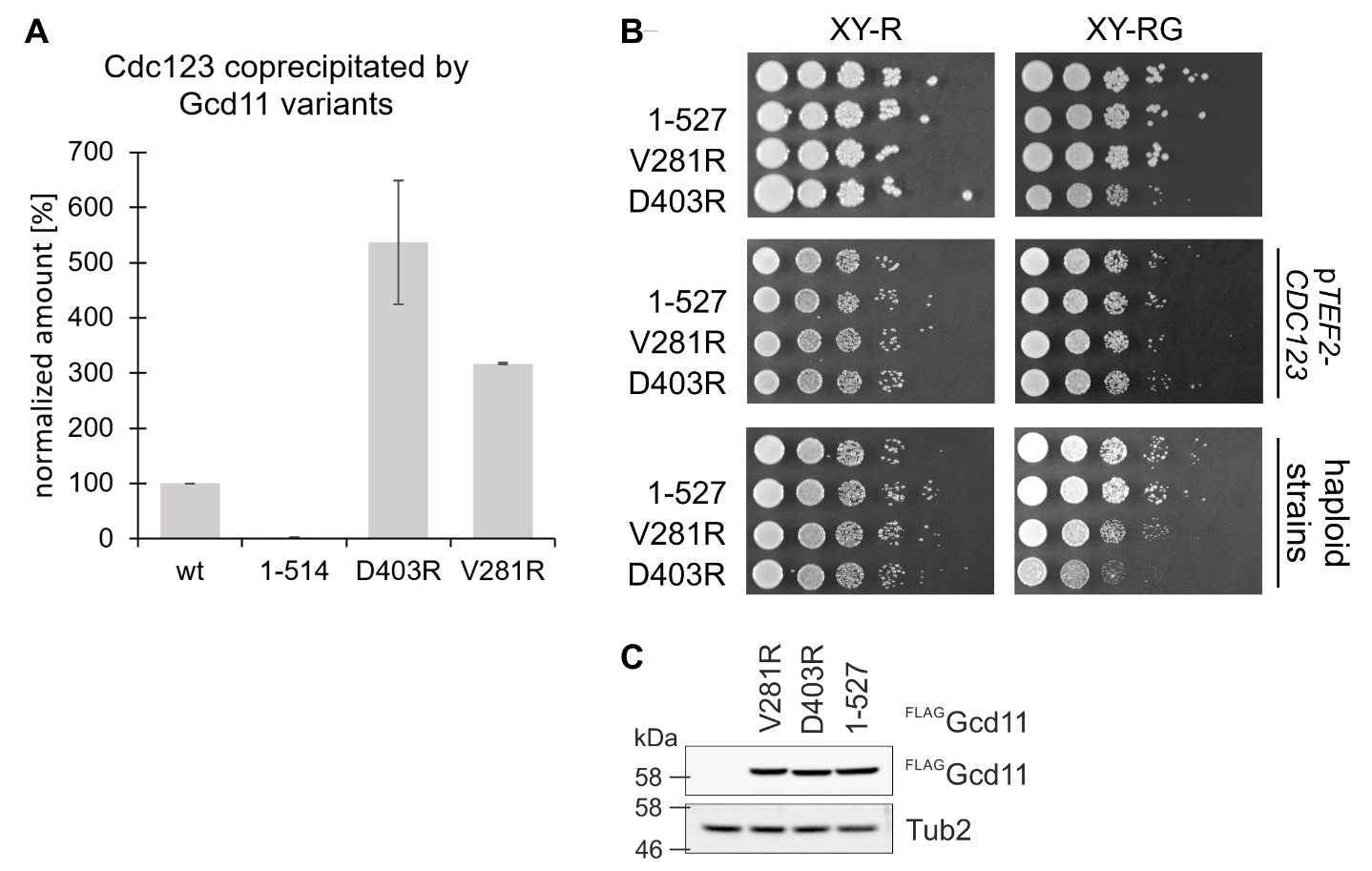 |
| --- |
| Supplemental Figure 1. **Effect of assembly incompetent Gcd11 variants on cell viability.** A, Quantification of co-precipitated Cdc123 shown in Western blot analysis in Fig. 2 *B*. Signals of immunoprecipitated Gcd11 variants and co-precipitated Cdc123 were measured using Odyssey V3.0 software. Cdc123 signals were normalized to the amount of the corresponding Gcd11 derivatives. The co-precipitation of Cdc123 is shown as mean and S.D. and co-precipitation of Cdc123 by Gcd11(1-527) was set to 1 (n = 3). B, Growth of strains overexpressing mutated variants of Gcd11. FLAG-tagged *GCD11* variants were expressed under control of the *GALL* promotor. Cells were spotted in 10-fold dilution series on solid complete media (XY) containing raffinose (R) or raffinose + galactose (RG). Plates were incubated at 25°C. Growth test was performed in diploid cells with only endogenous *CDC123* (upper panels) and diploid strains overexpressing *CDC123* (p*TEF2*-*CDC123*, panels in the middle). The growth assay was also performed in haploid strains expressing Gcd11 variants (lower panels). C, Comparison of expression levels of Gcd11 derivatives. All constructs were expressed form the *GALL* promotor for 2 h in asynchronous cultures. Cells were harvested by centrifugation and processed for Western blot analysis. Tubulin (Tub2) served as a loading control. |

| 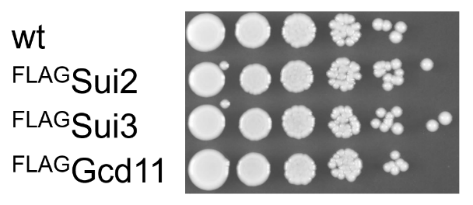 |
| --- |
| Supplemental Figure 2. **Viability of yeast strains with endogenously tagged eIF2 subunits.** On the left, the respective tagged subunit is indicated. Yeast cells were spotted in 10-fold dilution series on solid complete media (XY) containing glucose. Plates were incubated at 25°C for three days. |

| *Supplemental Table 1: Reporter plasmids, plasmids for E. coli and Flp-In™ T-REx™-293 cells used in this work* | | | |
| --- | --- | --- | --- |
| **Name** | **Insert** | **Vector** | **Figure** |
| pJG4-5 |  |  | 7A, B |
| pOG44 |  |  | 8C, D |
| pWS1898 | prha-HIS6-SUI3 | pJOE4056 | 6D |
| pWS1899 | prha-HIS6-SUI2 | pJOE4056 | 6D |
| pWS2015 | GCD11(310-527) | pEG202 | 7A, B |
| pWS3395 | GCN4(5’-UTR)-lacZ | pRS314 | 5E |
| pWS4126 | prha-GST-TEV | pJOE2955 | 6D |
| pWS4127 | prha-GST-TEV-CDC123 | pJOE2955 | 6D |
| pWS5495 | pCMV-FLAG3-EIF2S3-tbGH | pcDNA5-FRT-TO | 8B |
| pWS5590 | GCD11(1-309) | pJG4-5 | 7A, B |
| pWS5644 | pCMV-FLAG3-hCDCD123-tbGH | pcDNA5-FRT-TO | 8C |

| *Supplemental Table 2: Yeast strains used in this work, sorted by experiment* | | |  |  |
| --- | --- | --- | --- | --- |
| **Name** | ***MAT*** | **Genotype** | | **Figure** |
| K700 | α | *ade2-1, his3-11, 15, leu2-3, 112, trp1-1, ura3, ssd1delta, can1-100, [psi+], ho* | | 1B |
| W15011 | α | *leu2::pGAL1-FLAG3-GCD11(1-527)-tCYC1-LEU2* | |  |
| W14924 | α | *leu2::pGAL1-FLAG3-GCD11(AA61-527)-tCYC1-LEU2* | |  |
| W15009 | α | *leu2::pGAL1-FLAG3-GCD11(72-527)-tCYC1-LEU2* | |  |
| W15010 | α | *leu2::pGAL1-FLAG3-GCD11(81-527)-tCYC1-LEU2* | |  |
| W14925 | α | *leu2::pGAL1-FLAG3-GCD11(AA91-527)-tCYC1-LEU2* | |  |
| W14926 | α | *leu2::pGAL1-FLAG3-GCD11(AA140-527)-tCYC1-LEU2* | |  |
| W14927 | α | *leu2::pGAL1-FLAG3-GCD11(AA201-527)-tCYC1-LEU2* | |  |
| W14928 | α | *leu2::pGAL1-FLAG3-GCD11(AA310-527)-tCYC1-LEU2* | |  |
| W14929 | α | *leu2::pGAL1-FLAG3-GCD11(AA410-527)-tCYC1-LEU2* | |  |
| W12626 | a/α | *GCD11/gcd11-delta::kanMX6* | | 2B, 2E, S1A |
| W12783 | a/α | *GCD11/gcd11-delta::kanMX6 his3/his3::pGCD11-FLAG3-GCD11-tCYC1-HIS3* | |  |
| W15419 | a/α | *GCD11/gcd11-delta::kanMX6 his3/his3::pGCD11-FLAG3-GCD11(1-514)-tCYC1-HIS3* | |  |
| W15558 | a/α | *GCD11/gcd11-delta::kanMX6 his3/his3::pGCD11-FLAG3-GCD11(D403R)-tCYC1-HIS3* | |  |
| W15416 | a/α | *GCD11/gcd11-delta::kanMX6 his3/his3::pGCD11-FLAG3-GCD11(V281R)-tCYC1-HIS3* | |  |
| W15500 | a/α | *his3/his3::HIS3 GCD6/GCD6-HA3-KanMX6* | | 2C |
| W15512 | a/α | *his3/his3::pGCD11-FLAG3-GCD11-tCYC1-HIS3 GCD6/GCD6-HA3-KanMX6* | |  |
| W15506 | a/α | *his3/his3::pGCD11-FLAG3-GCD11(1-514)-tCYC1-HIS3 GCD6/GCD6-HA3-KanMX6* | |  |
| W15771 | a/α | *his3/his3::pGCD11-FLAG3-GCD11(D403R)-tCYC1-HIS3 GCD6/GCD6-HA3-KanMX6* | |  |
| W15503 | a/α | *his3/his3::pGCD11-FLAG3-GCD11(V281R)-tCYC1-HIS3 GCD6/GCD6-HA3-KanMX6* | |  |
| W15501 | a/α | *his3/his3::HIS3 GCD7/GCD7-HA3-KanMX6* | | 2D |
| W15513 | a/α | *his3/his3::pGCD11-FLAG3-GCD11-tCYC1-HIS3 GCD7/GCD7-HA3-KanMX6* | |  |
| W15507 | a/α | *his3/his3::pGCD11-FLAG3-GCD11(1-514)-tCYC1-HIS3 GCD7/GCD7-HA3-KanMX6* | |  |
| W15772 | a/α | *his3/his3::pGCD11-FLAG3-GCD11(D403R)-tCYC1-HIS3 GCD7/GCD7-HA3-KanMX6* | |  |
| W15504 | a/α | *his3/his3::pGCD11-FLAG3-GCD11(V281R)-tCYC1-HIS3 GCD7/GCD7-HA3-KanMX6* | |  |
| W10953 | a | *TIF5-HA3-HIS3MX6* | | 3A-D, S2 |
| W15228 | a | *kanMX4-pSUI2-FLAG3-SUI2 TIF5-HA3-HIS3MX6* | |  |
| W15230 | a | *kanMX4-pSUI3-FLAG3-SUI3 TIF5-HA3-HIS3MX6* | |  |
| W15232 | a | *kanMX4-pGCD11-FLAG3-GCD11 TIF5-HA3-HIS3MX6* | |  |
| W9878 | α | *SUI2-myc13-HIS3MX6* | | 3E, F |
| W14424 | α | *ura3::pTEF2-CDC123-FLAG3-tCYC1-URA3 SUI2-MYC13-HIS3MX6* | |  |
| K700 | α |  | | 4B |
| W15193 | α | *ura3::pGAL1-3xFLAG-SUI3-tCYC1-URA3* | |  |
| W15428 | α | *ura3::pGAL1-FLAG3-SUI3(YS/AA)-tCYC1-URA3* | |  |
| W15429 | α | *ura3::pGAL1-FLAG3-SUI3(LL/RR)-tCYC1-URA3* | |  |
| W15773 | a/α | *ura3/ura3::URA3 TIF5/TIF5-myc13-HIS3MX6* | | 4C |
| W15441 | a/α | *ura3/ura3::pGAL1-3xFLAG-SUI3-tCYC1-URA3 TIF5/TIF5-myc13-HIS3MX6* | |  |
| W15444 | a/α | *ura3/ura3::pGAL1-FLAG3-SUI3(YS/AA)-tCYC1-URA3 TIF5/TIF5-myc13-HIS3MX6* | |  |
| W15447 | a/α | *ura3/ura3::pGAL1-FLAG3-SUI3(LL/RR)-tCYC1-URA3 TIF5/TIF5-myc13-HIS3MX6* | |  |
| W5077 | a | *CDC123-MYC13-HIS3MX6* | | 4E |
| W15714 | a | *trp1::pTEF2-FLAG3-SUI2-tCYC1-TRP1 CDC123::CDC123-MYC13-HIS3MX6* | |  |
| W15720 | a | *trp1::pTEF2-FLAG3-SUI2(L205E)-tCYC1-TRP1 CDC123::CDC123-MYC13-HIS3MX6* | |  |
| W15723 | a | *trp1::pTEF2-FLAG3-SUI2(V220E)-tCYC1-TRP1 CDC123::CDC123-MYC13-HIS3MX6* | |  |
| K699 | a | *ade2-1 can1-100 his3-11 leu2-3,-112 trp1-1 ura3-2 ssd1* | | 5A |
| W14058 | a | *ura3::pTEF2-CDC123-FLAG3-tCYC1-URA3* | |  |
| W15415 | a | *ura3::pTEF2-CDC123-FLAG3-tCYC1-URA3 leu2::pGAL1-HA4-SUI2-tCYC1-LEU2* | |  |
| W15437 | a | *ura3::pTEF2-CDC123-FLAG3-tCYC1-URA3 leu2::pGAL1-HA4-SUI3-tCYC1-LEU2* | |  |
| W15910 | a/α | *ura3/ura3::pTEF2-CDC123-3xFLAG-tCYC1-URA3* | | 5B, C |
| W15911 | a/α | *SUI2/sui2delta::natNT2 ura3/ura3::pTEF2-CDC123-3xFLAG-tCYC1-URA3* | |  |
| K842 | a/α | *ade2-1/ade2-1 his3-11, 15/his3-11, 15 leu2-3,-112/leu2-3,-112 trp1-1/, trp1-1 ura3-2/ura3-2* | | 5D, E |
| W8907 | a/α | *SUI2/sui2delta::HIS3MX6* | |  |
| W9883 | a/α | *SUI3/sui3-delta::kanMX6* | |  |
| W12626 | a/α | *GCD11/gcd11-delta::kanMX6* | |  |
| K699 | a | *ade2-1, his3-11, 15, leu2-3, 112, trp1-1, ura3, ssd1delta, can1-100, [psi+], ho* | | 6B |
| W14058 | a | *ura3::pTEF2-CDC123-3xFLAG-tCYC1-URA3* | |  |
| W15914 | a | *ura3::pTEF2-CDC123-FLAG3-tCYC1-URA3 leu2::pGAL1-HA4-SUI2-tCYC1-LEU2* | |  |
| W15915 | a | *ura3::pTEF2-CDC123-FLAG3-tCYC1-URA3 leu2::pGAL1-HA4-SUI2(L205E)-tCYC1-LEU2* | |  |
| W15916 | a | *ura3::pTEF2-CDC123-FLAG3-tCYC1-URA3 leu2::pGAL1-HA4-SUI2(V220E)-tCYC1-LEU2* | |  |
| K699 | a | *ade2-1 can1-100 his3-11 leu2-3,-112 trp1-1 ura3-2 ssd1* | | 6C |
| W14058 | a | *ura3::pTEF2-CDC123-FLAG3-tCYC1-URA3* | |  |
| W15914 | a | *ura3::pTEF2-CDC123-FLAG3-tCYC1-URA3 leu2::pGAL1-HA4-SUI2-tCYC1-LEU2* | |  |
| W15915 | a | *ura3::pTEF2-CDC123-FLAG3-tCYC1-URA3 leu2::pGAL1-HA4-SUI2(L205E)-tCYC1-LEU2* | |  |
| W15916 | a | *ura3::pTEF2-CDC123-FLAG3-tCYC1-URA3 leu2::pGAL1-HA4-SUI2(V220E)-tCYC1-LEU2* | |  |
| W276 | α | *his3, trp1, ura3-52::URA3-lexA-op-lacZ,*  *leu2::pLEU2-lexA-op* | | 7A, B |
| W16901 | α | *his3, trp1, ura3-52::URA3-lexA-op-lacZ, leu2::pTEF2-CDC123-tCYC1* | |  |
| W17918 | a/α | *ura3/ura3::pMET3-HA3-GCD11(DII+III)-tCYC1 URA3 his3::HIS3* | | 7C |
| W17919 | a/α | *ura3/ura3::pMET3-HA3-GCD11(DII+III)-tCYC1 URA3 his3/his3::pGCD11-FLAG3-GCD11(DI)-tCYC1 HIS3* | |  |
| W17920 | a/α | *ura3/ura3::pMET3-HA3-GCD11(DII+III)-tCYC1 URA3 leu2/leu2::pTEF2-CDC123-tCYC1-LEU2 his3::HIS3* | |  |
| W17921 | a/α | *ura3/ura3::pMET3-HA3-GCD11(DII+III)-tCYC1 URA3 leu2/leu2::pTEF2-CDC123-tCYC1-LEU2 his3/his3::pGCD11-FLAG3-GCD11(DI)-tCYC1 HIS3* | |  |
| K700 | α | *ade2-1, his3-11, 15, leu2-3, 112, trp1-1, ura3, ssd1delta, can1-100, [psi+], ho* | | S1B, C |
| W15672 | α | *his3::pGALL-FLAG3-GCD11-tCYC1-HIS3* | |  |
| W16436 | α | *his3::pGALL-FLAG3-GCD11(V281R)-tCYC1-HIS3* | |  |
| W16441 | α | *his3::pGALL-FLAG3-GCD11(D403R)-tCYC1-HIS3* | |  |
| W17745 | a/α | *leu2/leu2::pTEF2-CDC123-tCYC1-LEU2 his3/his3::HIS3* | |  |
| W17746 | a/α | *leu2/leu2::pTEF2-CDC123-tCYC1-LEU2 his3/his3::pGALL-FLAG3-GCD11-tCYC1-HIS3* | |  |
| W17747 | a/α | *leu2/leu2::pTEF2-CDC123-tCYC1-LEU2 his3/his3::pGALL-FLAG3-GCD11(V281R)-tCYC1-HIS3* | |  |
| W17748 | a/α | *leu2/leu2::pTEF2-CDC123-tCYC1-LEU2 his3/his3::pGALL-FLAG3-GCD11(D403R)-tCYC1-HIS3* | |  |
| W19314 | a/α | *leu2/leu2::LEU2* | |  |
| W19315 | a/α | *his3::his3::pGALL-FLAG3-GCD11-tCYC1-HIS3 leu2/leu2::LEU2* | |  |
| W19316 | a/α | *his3/his3::pGALL-FLAG3-GCD11(V281R)-tCYC1-HIS3 leu2/leu2::LEU2* | |  |
| W19317 | a/α | *his3/his3::pGALL-FLAG3-GCD11(D403R)-tCYC1-HIS3 leu2/leu2::LEU2* | |  |

| *Supplemental Table 3: Mammalian cell lines* | |
| --- | --- |
| **Name** | **Figure** |
| Flp-In™ T-REx™-293 | 7A, B |
| Flp-In™ T-REx™-293_^FLAG^EIF2S3 | 7A |
| Flp-In™ T-REx™-293_^FLAG^CDC123 | 7B |

| *Supplemental Table 4: List of primary antibodies and their usage* | | | |
| --- | --- | --- | --- |
| **Antigen** | **Description** | **Dilution** | **Use in figure** |
| S. c. Cdc123 | rabbit serum, affinity purified, Davids Biotechnologie | 1:1000 | 1B, 2B, 3A-C, 4B, 4C, 7B |
| H. s. Cdc123 | rabbit serum, affinity purified, Davids Biotechnologie | 1:500 | 8A |
| H. s. EIF2S1 | rabbit polyclonal, Santa Cruz, FL-315 | 1:1000 | 8 |
| H. s. EIF2S2 | rabbit polyclonal, ProSci, 60-960 | 1:200 | 8 |
| H. s. EIF2S3 | rabbit polyclonal, Bioworld, A31100 | 1:200 | 8B |
| FLAG | mouse monoclonal, Thermo Scientific, M2 | 1:20000 | 1B, 2B, 3A-C, 4B-E, 7B |
| FLAG | rabbit polyclonal, Thermo Scientific, F7425 | 1:3000 | 1B, 2B, 3E, 5A, 5B, 6B, 6C |
| Gcd11 | rabbit serum, Davids Biotechnologie | 1:2500 | 3A, 3B, 3E, 4B, 4E, 5A, 5B, 6B, 6C |
| GST | mouse monoclonal, Santa Cruz, B14 | 1:4000 | 4F |
| HA | mouse monoclonal, cell culture supernatant, 12CA5 | 1:100 | 2C-D, 5A, 6C |
| HA | rabbit polyclonal, Santa Cruz, Y11 | 1:1000 | 6B, 7C |
| His | mouse monoclonal, Qiagen, tetra-His | 1:2000 | 4F |
| MYC | mouse monoclonal, cell culture supernatant, 9E10 | 1:100 | 3E, 4C |
| MYC | rabbit polyclonal, Santa Cruz, A14 | 1:500 | 4E |
| Sui2 | rabbit serum, Davids Biotechnologie | 1:1000 | 1B, 2B, 3A, 3B, 4B, 5B |
| Sui3 | rabbit serum, Davids Biotechnologie | 1:1000 | 1B, 2B, 3A, 3B, 3E, 4E, 5B, 6B, 6C |

| *Supplemental Table 5: List of secondary antibodies and their usage* | | |
| --- | --- | --- |
| **Name** | **Manufacturer** | **Dilution** |
| α-rabbit-IRDye®800CW | Li-COR Biosciences | 1:15000 |
| α-rabbit-IRDye®680CW | Li-COR Biosciences | 1:15000 |
| α-mouse-IRDye®800CW | Li-COR Biosciences | 1:15000 |
| α-mouse-IRDye®680CW | Li-COR Biosciences | 1:15000 |
